# Supplementary material for: Mapping long-read sequences to a complete reference genome reveals uncaptured variants across the β-Globin cluster in Africans with sickle cell disease
Source: HGG Adv. 2026 Sep 4;7(4):100669. doi: 10.1016/j.xhgg.2026.100669 (PMC13651657; doi:10.1016/j.xhgg.2026.100669)
Supplement: Supplementary file 1 [file mmc1.pdf]

HGGA, Volume *7*

Supplemental information

Mapping long-read sequences to a complete reference genome reveals uncaptured variants across the β-Globin cluster in Africans with sickle cell disease

Pamela Lurie, Medhat Mahmoud, Aparna Haldipur, Yixing Han, Qing Li, Edmond Wonkam Tingang, Natalie Asmus, Emilyn Banfield, Nancy J. Hall, Bamidele Tayo, Richard Cooper, Titilola Akingbola, George Ayodo, Stephen Obaro, Guillaume Lettre, Qing Meng, Shalini Jhangiani, R. Alan Harris, Kim C. Worley, Juyhun Kim, Arang Rhie, Adam M. Phillippy, Fritz Sedlazeck, Ambroise Wonkam, and Neil A. Hanchard

SUPPLEMENTAL MATERIALS AND METHODS

Supplemental Methods

Restriction Fragment Length Polymorphism Haplotyping

Briefly, five gene regions within the beta-globin cluster on chromosome 11 were amplified by PCR using GoTaq polymerase and buffer (Promega, USA), Deoxyribonucleotide triphosphate master mix;(Fermentas, USA), and forward and reverse primers (Eurofins MWG Operon) under the PCR cycling conditions given in **Table S2.** Amplified DNA (1ul) was then loaded onto a 2% agarose gel in 1X TAE buffer with 0.5 micrograms/mL ethidium bromide and run at 135V for 40 to 50 minutes before inspecting for the expected product band size. The appropriately amplified product was then digested according to the manufacturer's instructions by combining 10ul of DNA with a master mix containing the cut enzyme (Fermentas, USA) and Tango buffer (Fermentas, USA). The mix was left in the PCR thermocycler to incubate overnight at 37°C. Subsequently, the mix was immediately placed on ice until loaded into a 3% agarose gel with 1X TAE buffer with 0.5 micrograms/mL ethidium bromide and run at 160V for 40 – 50 minutes. Digestion products were scored as +/+ if both alleles were digested, as +/– if only one allele was digested (heterozygote), and as –/– if no digestion was noted. Samples were then classified as one of the common RFLP sickle haplotypes **(Table S2)**.

Supplemental Tables

Table S1 - Demographic distribution of SCD samples. ID - internal deidentified ID; Haplotypes - RFLP ascertained sickle haplotype; Origin - city or region of recruitment; *Louisiana, USA[41].

Country	Cases	Haplotypes	Ancestry Group	Origin	HbF
Nigeria	SC004	Benin	Yoruba	Ibadan	n/a
	SC006	Benin/Atypical	Yoruba	Ibadan	2.6
	SC026	Atypical	Yoruba	Ibadan	2.8
	347	Benin	Hausa		7.3
	467	Benin/Atypical	Hausa		11.2
	886	CAR/Benin	Hausa		26.4
	898	Benin	Hausa		1.3
	942	Benin	Hausa		5.6
	SC097	Benin	Igbo	Ibadan	6.8
	SC144	Benin	Igbo	Ibadan	5.2
	SC148	Benin	Igbo	Ibadan	1.6
Cameroon	476	Cameroon	Mbamois	Douala	0.8
	41	Benin	Bami	Douala	10.9
	15	Benin/Cameroon	bakoko/Dla	Douala	0.9
	21	Benin/Cameroon	Beti	Yaoundé	23.4
	38	Benin	Beti	MBALMAYO	13
	40	Benin/Cameroon	Beti	Yaoundé	12
	144	Benin	Beti	Yaoundé	n/a
Kenya	9215	CAR	Luo	Lwak	11.1
	9218	CAR	Luo	Lwak	2.3
	9220	CAR	Luo	Lwak	6.4
	9238	CAR	Luo	Lwak	3.1
	9239	CAR	Luo	Lwak	7.1
	9242	CAR	Luo	Lwak	2.6
	9250	Benin/Cameroon	Luo	Lwak	7.3
	9246	CAR	Luo	Lwak	6.1
	9258	CAR	Luo	Lwak	1.5
	9263	CAR/Cameroon	Luo	Lwak	6.6
	9287	CAR/Cameroon	Luo	Lwak	0.7
	9292	CAR	Luo	Lwak	6.2
	9299	CAR	Luo	Lwak	3.4
	9296	Senegal	Luo	Lwak	17.8
	9295	Senegal	Luo	Lwak	7.8
	2169	CAR	Luo	Lwak	12.5
USA	NR134	Senegal	English	Louisiana	n/a
	NR012	Senegal	English	Louisiana	n/a
	NR039	Senegal	English	Louisiana	n/a
	R014	CAR/Benin	English	Louisiana	n/a
	R116	CAR/Benin	English	Louisiana	n/a
	R26	Senegal	English	Louisiana	n/a

Table S2 - RFLP haplotype and thermal cycling parameters (upper) alongside expected cleavage patterns for RFLP haplotypes (lower).

GENE REGION	PRIMER SEQUENCE		PCR THERMAL CYCLING CONDITIONS	FRAGMENT SIZE (bp)
	FORWARD	REVERSE		
5'Gγ	AACTGTTGCTTT ATAGGATTTT	TAGGAGCTTATTGA TAACCTCAGAC	1 cycle of 95 °C for 5min	657
			35 cycles of 94°C for 30sec, 53°C for 40sec, 72°C for 2min	
			1 cycle of 72°C for 7min<br>1 cycle of 4°C forever	
Gγ/Aγ1	TGCTGCTAATGC TTCATTACAA	AAGTGTGGAGTGTG CACATGA	1 cycle of 95 °C for 5min	780
			35 cycles of 94°C for 30sec, 53°C for 40sec, 72°C for 2min	
			1 cycle of 72°C for 7min<br>1 cycle of 4°C forever	
Gγ/Aγ2	TGCTGCTAATGC TTCATTACAA	TAAATGAGGAGCA TGCACACAC	1 cycle of 95 °C for 5min	780
			35 cycles of 94°C for 30sec, 53°C for 40sec, 72°C for 2min	
			1 cycle of 72°C for 7min<br>1 cycle of 4°C forever	
3'ψβ	TCTGCATTTGAC TCTGTTAGC	GGACCCTAACTGAT ATAACTA	1 cycle of 95 °C for 5min	614
			35 cycles of 94°C for 30sec, 55°C for 40sec, 72°C for 2min	
			1 cycle of 72°C for 7min<br>1 cycle of 4°C forever	
5'β	CTACGCTGACCT CATAAATG	CTAATCTGCAAGAG TGTCT	1 cycle of 95 °C for 5min	386
			30 cycles of 94°C for 30sec, 55°C for 40sec, 72°C for 40sec	
			1 cycle of 72°C for 7min<br>1 cycle of 4°C forever	

	XmnI<br>5'Gg	*HindIII*<br>Gg_1	*HindIIIA*<br>Gg_2	*HincII*<br>3b	*Hinfl*<br>5b
Senegal	+	+	-	+	+
Bantu/CAR	-	+	-	-	-
Cameroon	-	+	+	+	-
Benin	-	-	-	+	-
Arabic-Indian	+	+	-	+	-
Atypical	-	-	-	+	+

Table S3 - Long-read sequencing primers. Primers used to amplify beta-globin cluster for long-read sequencing.

	SEQUENCE	LEN	Tm	Dimer/ Secondary	GC%	E-VALUE	HG38 browser coordinates (Chr11)	Lift over from HG38 to T2T coordinates
AMP1	GTCAGGTGGTCAGCTTC (Forward)	17	56.4	No/No	58%	0.016	11:5280633-5280649	chr11:5339993-5340009
	TAAGTGCTCAGGGCTGTGTG (Reverse)	20	64.1	No/No	55%	0.008	11:5290195-5290214	chr11:5349548-5349567
AMP 2	GAGAGTCACCAGGTCAGT (Forward)	18	54.6	No/No	55%	0.083	11:5267198-5267215	chr11:5326554-5326571
	ATGAAGGCAGTGGCCCTT (Reverse)	18	64.7	No/Mod.	55%	0.083	11:5272969-5272986	chr11:5332331-5332348
AMP3	AATCTGCCATGGGAAGG (Forward)	17	61.1	No/No	53%	0.64	11:5257393-5257409	chr11:5316750-5316766
	TATGTCTTTCTCACCCCACCT (Reverse)	21	62.3	No/No	48%	0.042	11:5267310-5267330	chr11:5326666-5326686
AMP 4	ACGCTGATGCTGACTTGTGA (Forward)	20	64.8	No/No	50%	0.49	11:5252429-5252448	chr11:5311785-5311804
	CCCCTCAACATCACGTCCTT (Reverse)	20	66.2	No/No	55%	0.49	11:5257901-5257920	chr11:5317258-5317277
AMP10	ACCGGTCAAAGGCCATAGTG (Forward)	20	66	No/No	55%	0.016	11:5200307-5200326	chr11:5259660-5259679
	GTCTCATGCCAGAACCT (Reverse)	17	55.8	No/Weak	53%	0.64	11:5208290-5208306	chr11:5267638-5267654
AMP 5	CGCTCCAGGAAAAGTGACCT (Forward)	20	65	No/Weak	55%	0.008	11:5245566-5245585	chr11:5304931-5304950
	TTCTTCGTTGAGCCCCTTCC (Reverse)	20	67.5	No/No	55%	0.008	11:5252809-5252828	chr11:5312165-5312184
AMP 6	TAACATCTAGATTTCTGCACTGT (Forward)	20	57.2	No/Very Weak	35%	0.0004	11:5239005-5239027	chr11:5298378-5298400
	GACGAGGGGAGCTTTGCAAA (Reverse)	20	68.6	No/No	555	0.016	11:5247078-5247097	chr11:5306443-5306462
AMP 7	AGAGCTGCGGAGAAGAGGTA (Forward)	20	63.2	No/No	55%	0.008	11:5233089-5233108	chr11:5292462-5292481
	AGCACATAGATAGGCGGTG (Reverse)	19	60.3	No/Weak	53%	0.008	11:5240318-5240336	chr11:5299691-5299709
AMP 8	AGTTCCGGGAGACTAGCACT (Forward)	20	61.8	No/Very Weak	55%	0.008	11:5224887-5224906	chr11:5284255-5284274
	ACATGGGTATGGGAGAGGC (Reverse)	19	63.8	No/Very Weak	58%	0.021	11:5233652-5233670	chr11:5293025-5293043
AMP 9	TCGTGACTGAATTTTACCTT (Forward)	20	56.3	No/No	35%	0.008	11:5222580-5222599	chr11:5281948-5281967
	CCAGGCCATCACTAAAG (Reverse)	17	56.5	No/No	53%	0.33	11:5226663-5226679	chr11:5286031-5286047
AMP 11	CTACTGCCCAATCCTCCGTC (Forward)	20	65.8	No/No	60%	0.008	11:5595129-5595148	chr11:5654534-5654553
	ATAGTGAGGCGGCTCAGAGA (Reverse)	20	64	No/Mod.	55%	0.008	11:5603541-5603560	chr11:5662975-5662994

Table S4 - Barcode primers (Pacific Biosciences, California) for amplicon sequencing.

PRIMER ID	FORWARD SEQUENCE		PRIMER ID	REVERSE SEQUENCE
_0097_Forward	TACGATCGTAGCTGCT		_0097_Reverse	AGCAGCTACGATCGTA
_0098_Forward	TATACACACTCGCTCG		_0098_Reverse	CGAGCGAGTGTGTATA
_0099_Forward	AGCGCTGCGACACGCG		_0099_Reverse	CGCGTGTCGCAGCGCT
_0100_Forward	GTCGTAGCTGCTGTAT		_0100_Reverse	ATACAGCAGCTACGAC
_0101_Forward	CTGTACTAGAGCGTCT		_0101_Reverse	AGACGCTCTAGTACAG
_0102_Forward	TCGAGTGTATAGCTCA		_0102_Reverse	TGAGCTATACACTCGA
_0103_Forward	ACTGTGACAGTATGAT		_0103_Reverse	ATCATACTGTCACAGT
_0104_Forward	TGTCTGAGACGCATAC		_0104_Reverse	GTATGCGTCTCAGACA
_0105_Forward	CACTCACGTGTGATAT		_0105_Reverse	ATATCACACGTGAGTG
_0106_Forward	ATCGCATCGCAGAGAC		_0106_Reverse	GTCTCTGCGATGCGAT
_0107_Forward	TACTCATATATGCTAC		_0107_Reverse	GTAGCATATATGAGTA
_0108_Forward	GTCTACGCTCGTCGCG		_0108_Reverse	CGCGACGAGCGTAGAC
_0109_Forward	TGCGAGACTATCGCGA		_0109_Reverse	TCGCGATAGTCTCGCA
_0110_Forward	CAGATCTCTCTGATGT		_0110_Reverse	ACATCAGAGAGATCTG
_0111_Forward	GTAGAGTGATCGCGTC		_0111_Reverse	GACGCGATCACTCTAC
_0112_Forward	ACGACAGTCAGAGTAT		_0112_Reverse	ATACTCTGACTGTCGT
_0113_Forward	ATATATAGCTGATGCG		_0113_Reverse	CGCATCAGCTATATAT
_0114_Forward	TGCTATCTGAGATACT		_0114_Reverse	AGTATCTCAGATAGCA
_0115_Forward	CAGCAGATCATGTCGA		_0115_Reverse	TCGACATGATCTGCTG
_0116_Forward	TGCTGCGAGCGCTCTG		_0116_Reverse	CAGAGCGCTCGCAGCA
_0117_Forward	ACTATCGCAGCTCAGT		_0117_Reverse	ACTGAGCTGCGATAGT
_0118_Forward	CGTCTCTCGTCTGTGC		_0118_Reverse	GCACAGACGAGAGACG
_0119_Forward	GAGTCTCGATATACTA		_0119_Reverse	TAGTATATCGAGACTC
_0120_Forward	TGTCATGTGTACACAC		_0120_Reverse	GTGTGTACACATGACA

_0121_Forward	TCTGTCGATATACACT		_0121_Reverse	AGTGTATATCGACAGA
_0122_Forward	ACGTGCTCTATAGAGA		_0122_Reverse	TCTCTATAGAGCACGT
_0123_Forward	TATCAGCACGACATGC		_0123_Reverse	GCATGTCGTGCTGATA
_0124_Forward	GCTCTCACGATATCAG		_0124_Reverse	CTGATATCGTGAGAGC
_0125_Forward	TATATGCTCTGTGTGA		_0125_Reverse	TCACACAGAGCATATA
_0126_Forward	GATAGCTGCTAGCTGA		_0126_Reverse	TCAGCTAGCAGCTATC
_0127_Forward	TCTCATGTGTGAGCTA		_0127_Reverse	TAGCTCACACATGAGA
_0128_Forward	TCAGATGTGTCGCGAG		_0128_Reverse	CTCGCGACACATCTGA
_0129_Forward	CGTAGCTCAGACACTC		_0129_Reverse	GAGTGTCTGAGCTACG
_0130_Forward	TCAGAGACACTACGAG		_0130_Reverse	CTCGTAGTGTCTCTGA
_0131_Forward	ATCGAGCAGCAGTCGT		_0131_Reverse	ACGACTGCTGCTCGAT
_0132_Forward	CGTAGCTCGAGATGAG		_0132_Reverse	CTCATCTCGAGCTACG
_0133_Forward	GCTAGTCGATGACAGC		_0133_Reverse	GCTGTCATCGACTAGC
_0134_Forward	CATGATGCGAGACGCT		_0134_Reverse	AGCGTCTCGCATCATG
_0135_Forward	GTGTAGCGTAGACAGT		_0135_Reverse	ACTGTCTACGCTACAC
_0136_Forward	AGCACGTGTGTCGACA		_0136_Reverse	TGTCGACACACGTGCT

Table S5 - **Read lengths.** Raw read lengths and Canu trimmed contig read lengths per amplicon.

		Raw Reads		Canu contigs	
	expected length	avg read length	read length distribution	avg contig length	contig length distribution
amp1	9,581	6578	50 - 87967	7161.4	407 - 13917
amp2	5,788	6917	50 - 97123	6346.1	426 - 13995
amp3	9937	6942	50 - 72850	8076.7	439 - 18489
amp4	5,491	8301	50 - 76142	8207.1	412 - 22278
amp5	7,262	7017	50 - 101191	8088.8	416 - 21880
amp6	8,092	6972	50 - 86806	8410.7	414 - 24200
amp7	7,247	7876	50 - 76065	9018	444 - 29330
amp8	8,783	7687	50 - 116525	6899.3	471 - 8815
amp9	4,102	7463	50 - 72910	4100	424 - 10256

Table S7. Sample cohorts for all 2,800 participants with lrWGS data. Table adapted from All of Us user support hub online documentation Table 16, https://support.researchallofus.org/hc/en-us/articles/29475228181908-How-the-All-of-Us-Genomic-data-are-organized#01JJK689B1VNXW0KFJ3JC1X0N6. 42 CDRv8 participants were sequenced in two different sample batches.

**Cohort name**	**Sequencing facility**	**Sequencing platform**	**No. of samples**	**Samples of African ancestry**	**Minimum coverage**
HA_Seq_CDRv7	HA	PacBio Sequel IIe and Sequel II	1027	959	Mid-pass (12x)
HA_Rev_mid	HA	PacBio Revio	65		Mid-pass (12x)
BI_Seq_high	BI	PacBio Sequel IIe	84	16	High-pass (25x)
BI_Seq_mid	BI	PacBio Sequel IIe	198	46	Mid-pass (12x)
BI_Rev_mid	BI	PacBio Revio	803	191	Mid-pass (12x)
BCM_Seq_high	BCM	PacBio Sequel IIe	77	8	High-pass (25x)
BCM_Rev_high	BCM	PacBio Revio	111	10	High-pass (25x)
BCM_ONT_high	BCM	ONT R10.4 on PromethION	196	20	High-pass (25x)
JHU_ONT_high	JHU	ONT R10.4 on PromethION	128		High-pass (25x)
UW_Seq_high	UW	PacBio Sequel IIe	100		High-pass (25x)
UW_Rev_high	UW	PacBio Revio	53		High-pass (25x)
**Total samples**			**2842**	**1250**	

Table S9. Common structural variants (SV). CHR= chromosome; POS = reference start position in basepairs ((T2T.CHM13.v2); END =end position in basepairs; NUM = Number of individuals with called SV at the same location (SURVIVOR2). ANNO= nearest gene annotation in T2T.CHM13.v2; Conf SUPP = number of supporting genotype calls exactly matching SV call; AVE LEN= Average length; CI START | CI END = confidence intervals around potential start and end positions upstream (up-) and downstream (down-) relative to reference.

**CHR**	**POS**	**END**	**SV**	**ANNO**	**NUM**	**LEN (BP)**	**CI START (**up-)	**CI START (**down-)	**CI END** (up-)	**CI END** (down-)	**Conf SUPP**
chr11	5259660	5267655	DUP	*OR51V1*	6	7,995	-1	6	-2	8	6
chr11	5286643	5286643	INS	*HBB*	5	8,563	-1,805	4	-1,805	4	5
chr11	5293620	5293680	INV	*HBD*	9	75	0	571	-23	565	3
chr11	5295149	5295149	INS	Up- or in *HBD*	4	4,044	-219	0	-219	0	4
chr11	5297452	5297508	INV	Up- or in *HBD*	10	60	-2	260	-17	282	4
chr11	5299784	5299838	INV	Up- or in *HBD*	11	54	-76	0	-79	0	5
chr11	5317572	5317811	INV	Down- of *HBE1*	30	196	-470	960	-113	761	8
chr11	5324507	5324556	INV	Down- of *HBE1*	5	57	-592	0	-603	0	1
chr11	5330321	5330321	INS	*HBE1*	4	25,212	-1,889	0	-1,889	0	4
chr11	5341138	5341283	INV	*HBE1*	13	150	-305	0	-291	8	1
chr11	5340540	5348414	DEL	*HBE1*	17	-7,859	0	105	0	0	7
chr11	5343104	5343150	INV	*HBE1*	4	104	-883	0	-705	0	1
chr11	5345463	5345463	INS	*HBE1*	4	2,558	0	621	0	621	3
chr11	5346603	5346720	INV	*HBE1*	5	96	0	1,581	0	1,513	1

Supplemental Figures

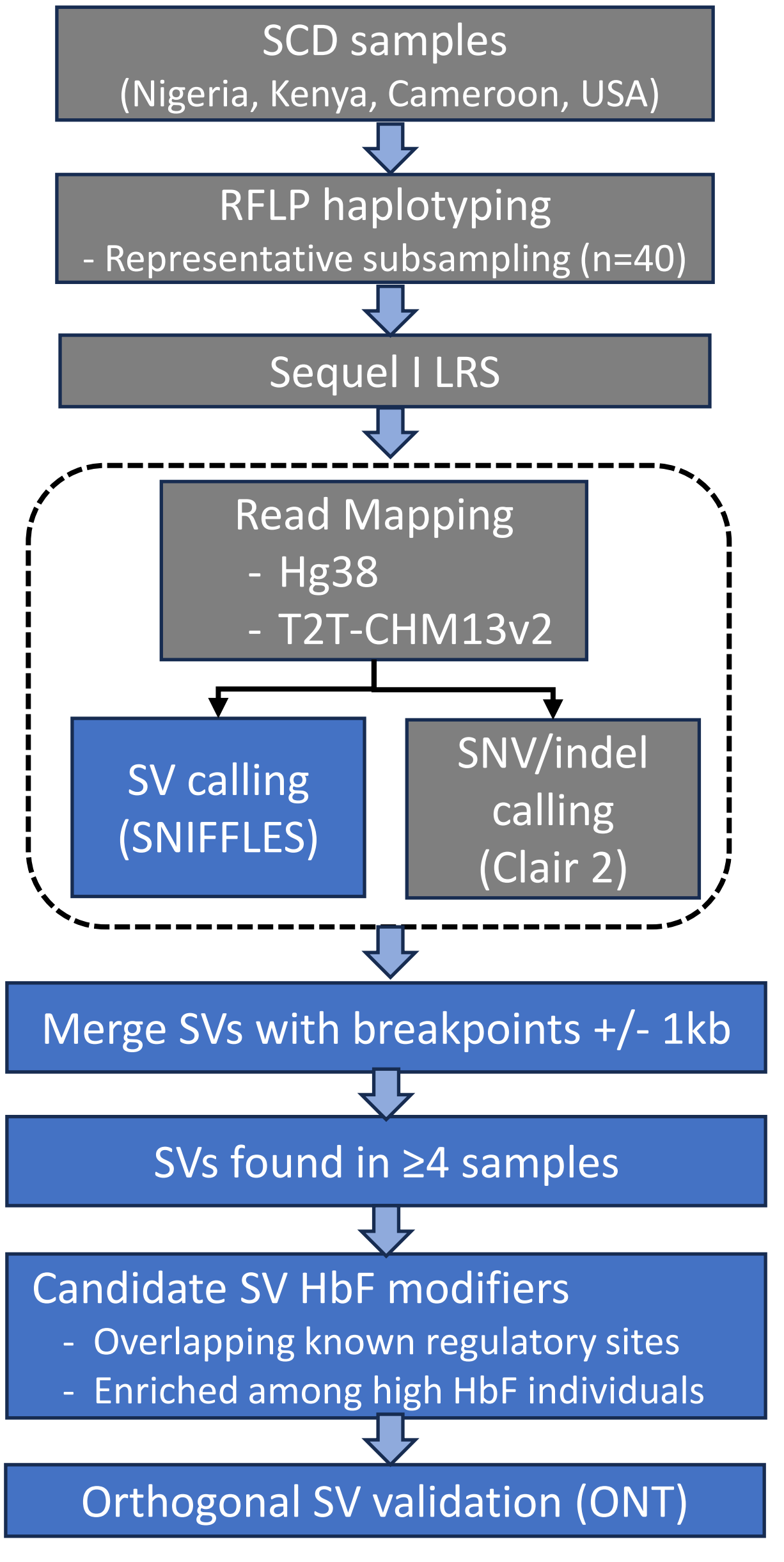

Figure S1. Study overview. DNA samples from SCD participants representative of classical SCD restriction fragment length (RFLP) haplotypes underwent long-read sequencing (LRS), prior to mapping reads to two human genome references and calling structural variants (SVs) and single nucleotide variants (SNVs) in PRINCESS (dotted line). SV calls were then prioritized for downstream evaluation.

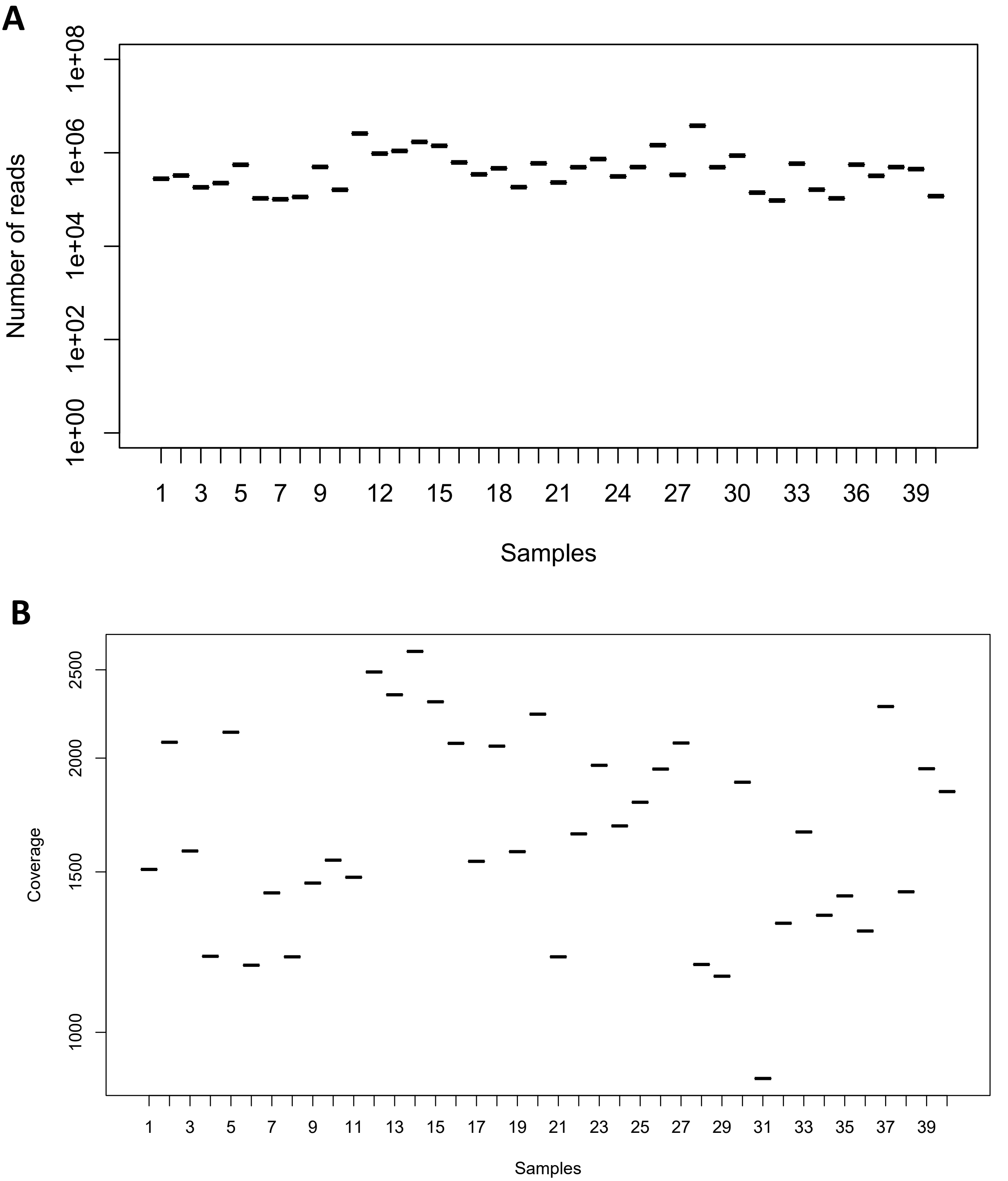

Figure S2. Long-read sequence coverage for samples. A. shows the total number of reads obtained for each sample. **B.** shows the median depth of coverage for each individual across the region.. Base pair coverage of the overall beta-globin locus in the T2T-CHM13.v2 reference genome ranged from +100bp - >90,000kb.

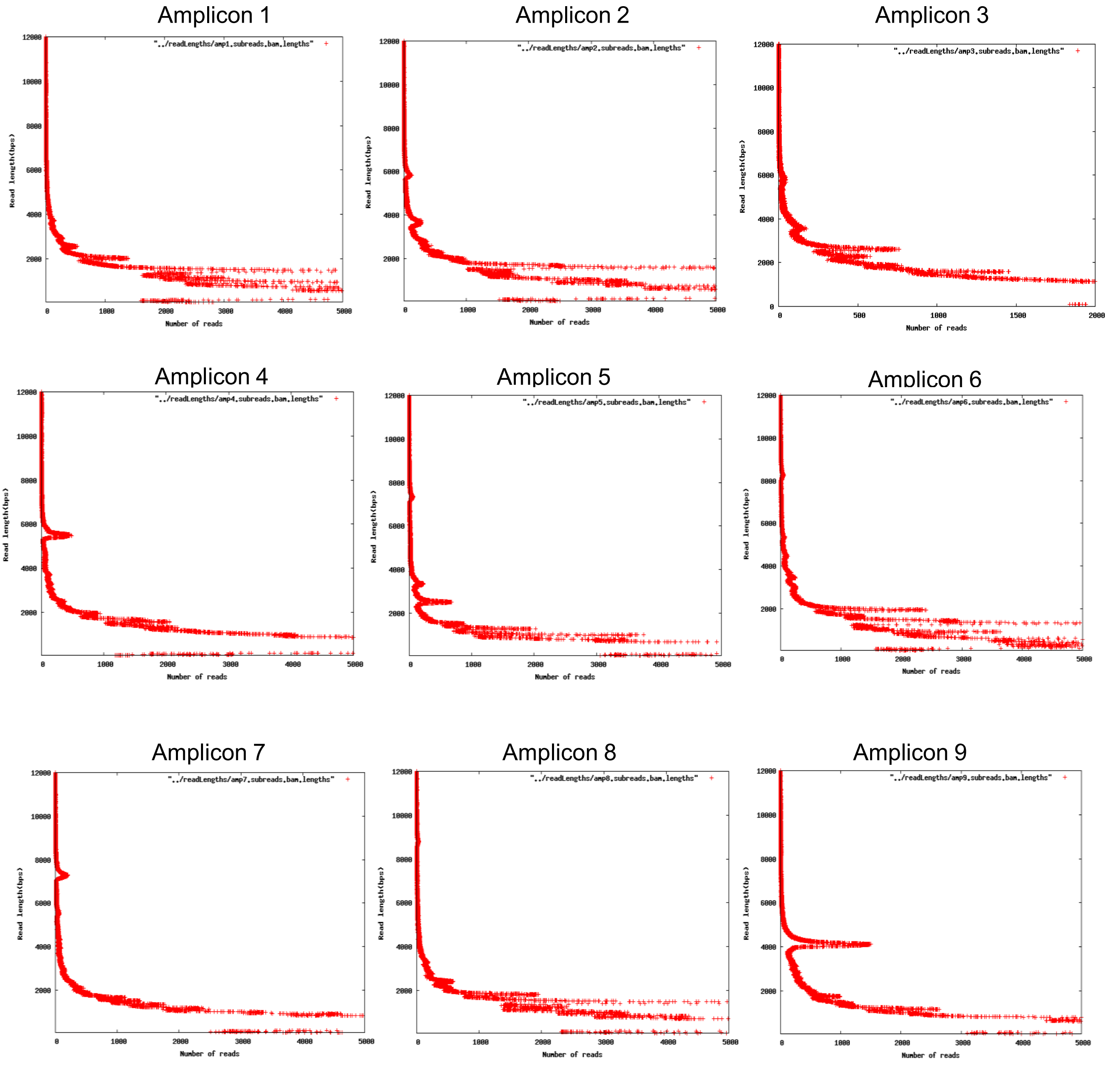

Figure S3. Distribution of raw reads for each gene-based amplicon sequenced.

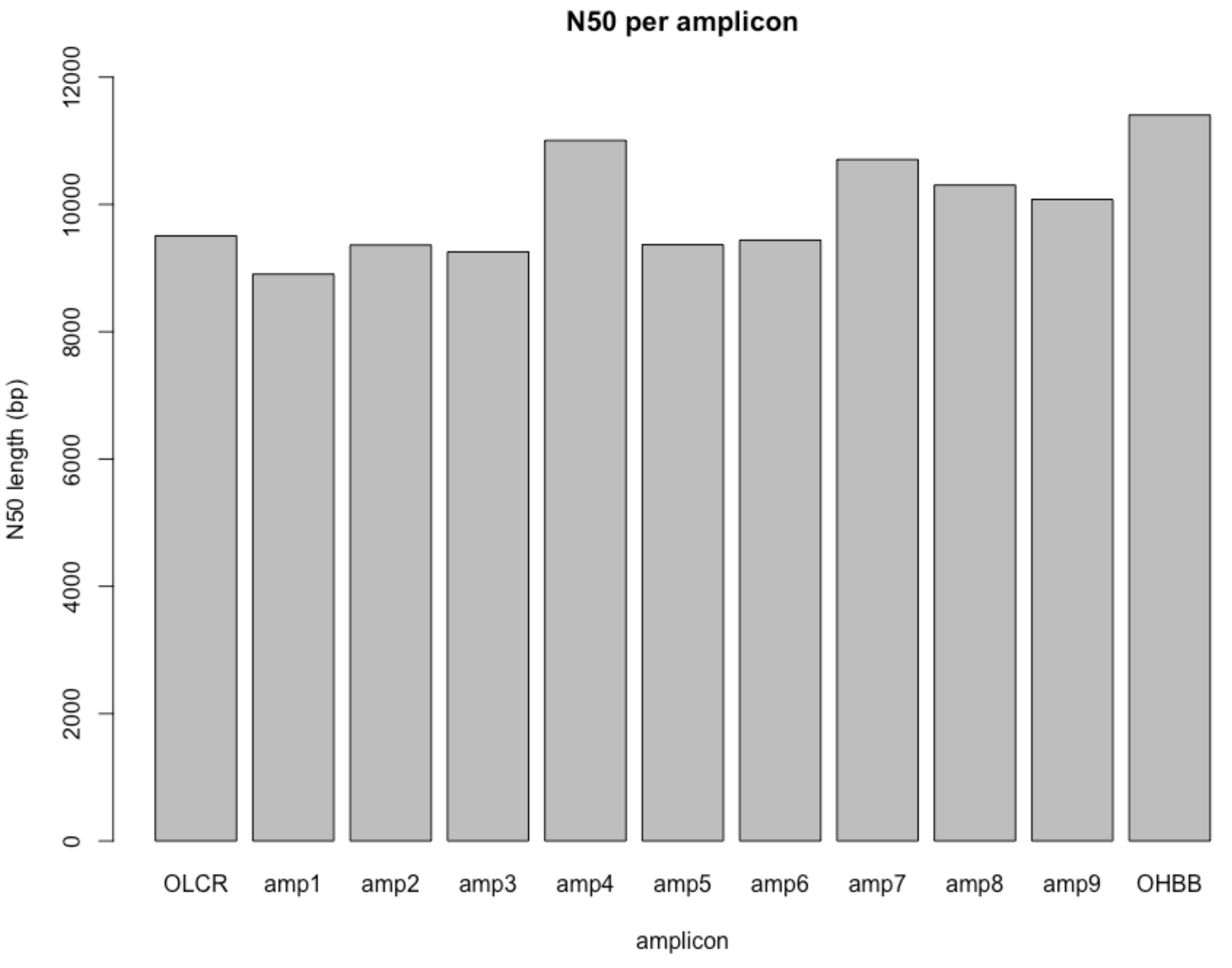

Figure S4. Read length N50 per amplicon from raw reads. Half of the amplicon is covered by reads larger than N50 length (expected read length exceeded).

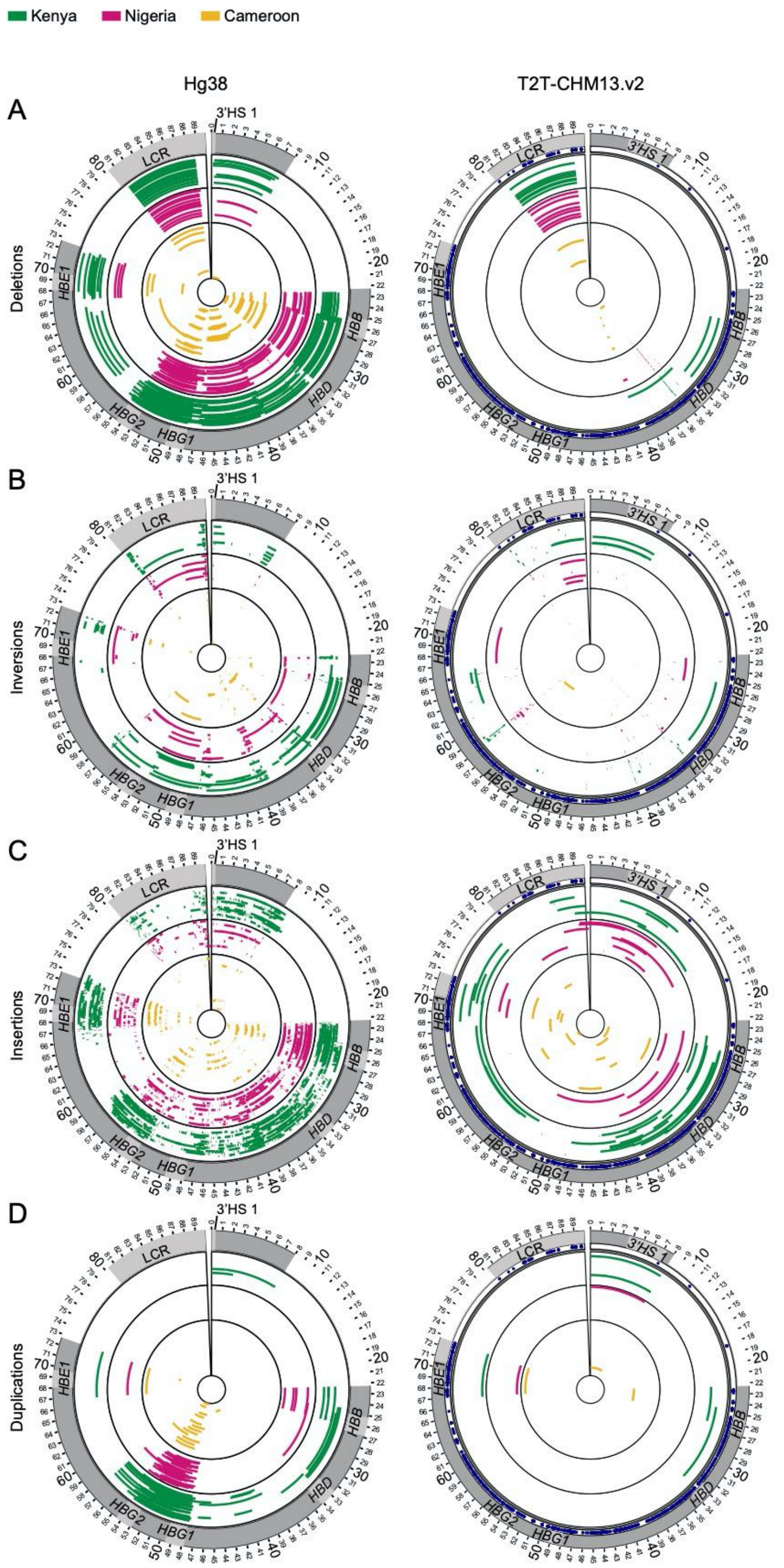

Figure S5. Genomic context of identified structural variants. Lighter gray shaded regions represent gene and Hypersensitive sites located in the beta-globin locus. Blue circles beneath the gene name and sequenced region track on T2T-CHM13.V2 circular plots represent areas of unalignment between the Hg38 and T2T-CHM13.v2 reference genomes. (**A**) Deletions. (**B**) Inversions. (**C**) Insertions. (**D**) Duplications. Geographical groups from Africa indicated by color: Kenya = green, Nigeria = pink, Cameroon = yellow. White spaces represent regions of the genome that was not sequenced. Note: 3' HS 1 location is further downstream *HBB* in T2T-CHM13.v2.

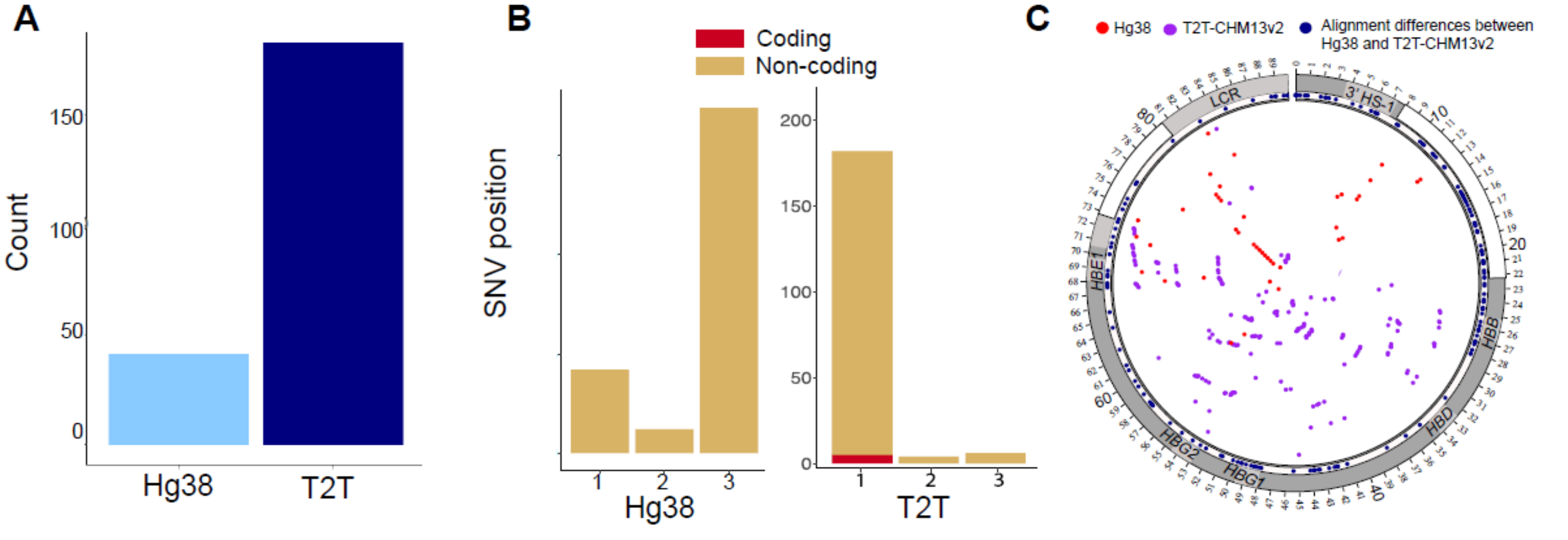

Figure S6. Single Nucleotide Variants (SNVs) in the beta-globin cluster of Africans with SCD. **A** - total number of novel SNVs called after aligning to either hg38 or T2T_CHM13.v2 (T2T) genome references. **B** – number of SNVs (X category) seen at each novel SNV position (Y axis); **C** – novel SNVs in genomic context across the beta-globin locus. Each concentric line from outside in represents an individual and each dot a different SNV in that individual. The outermost ring shows the relative genomic position in the region in kilobases (kbs). The next outermost ring illustrates non-coding (dark gray) or coding (lighter gray) targeted sequence; non-targeted regions are intervening in white. Navy blue dots in the next most outer ring represent DNA sequence found in T2T-CHM13.V2 but not in Hg38.

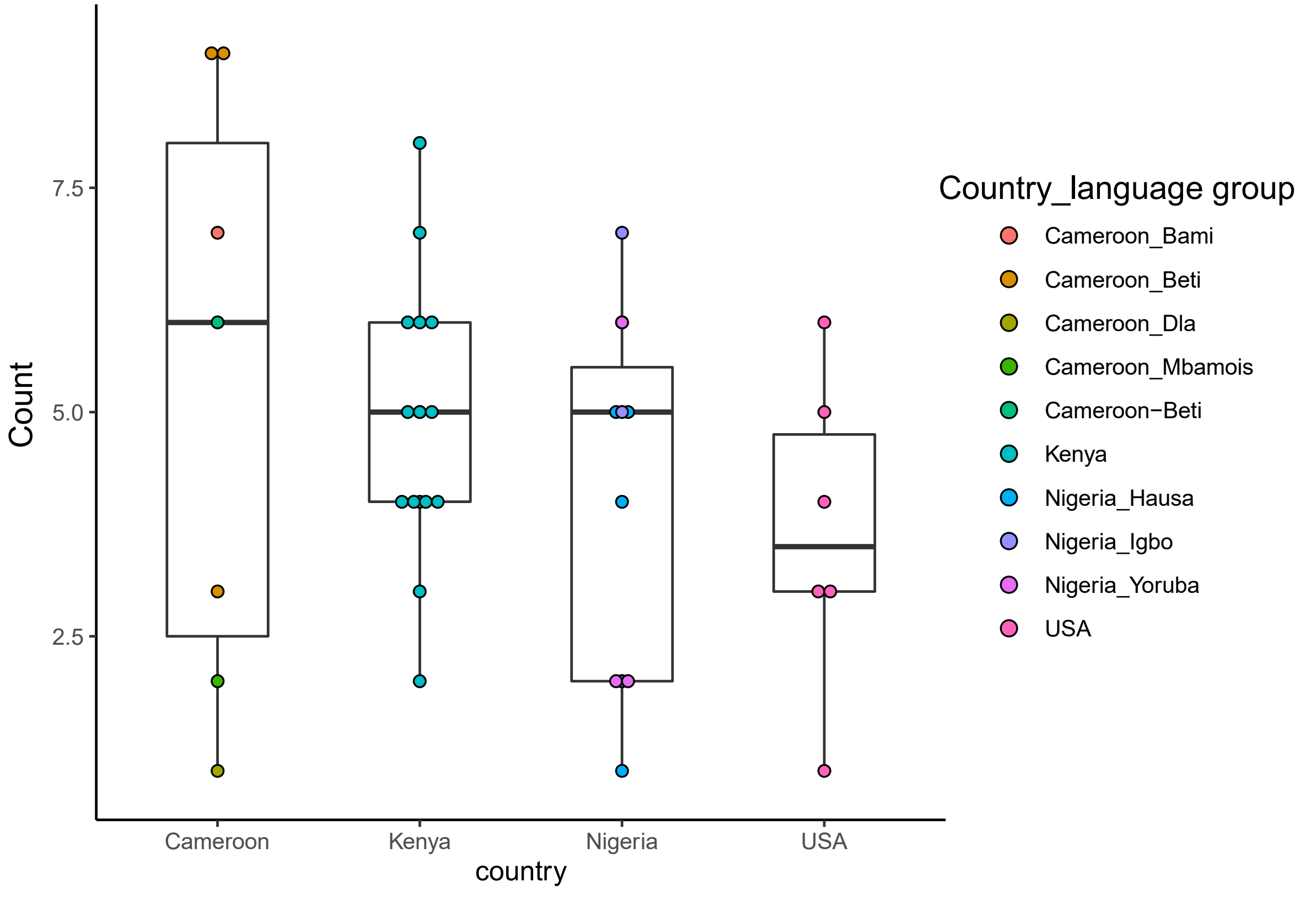

Figure S7. **Merged SVs per person grouped by geographical groups**. There is no significant difference between the median number of SVs per group

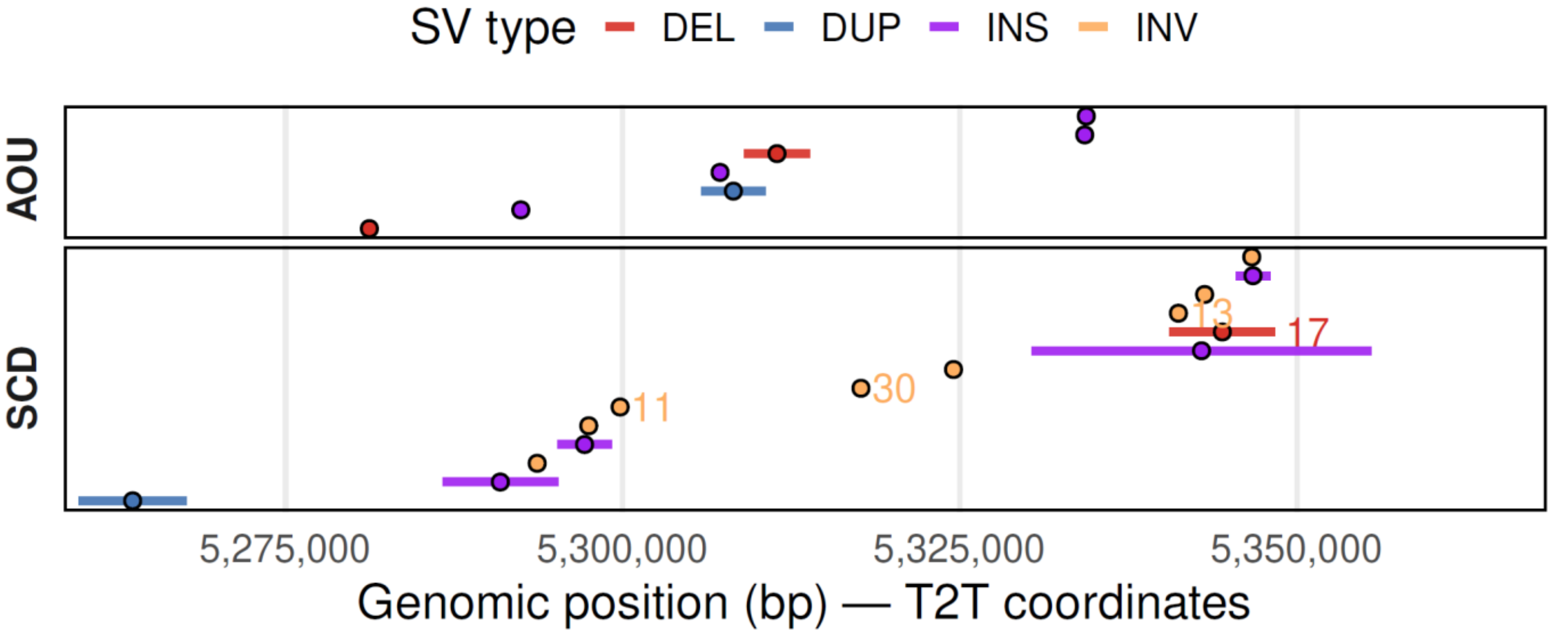

Figure S8. Structural variants (SVs) in African Sickle Cell Disease (SCD) participants and from long-read sequencing of All of Us (AoU) Africans. Numbers beside SVs are shown for SV shared by >10 samples. SV types – DEL=Deletion; DUP=Duplication; INS=Insertion; INV=inversion. Coordinates are referenced to T2T.CHM13v2.

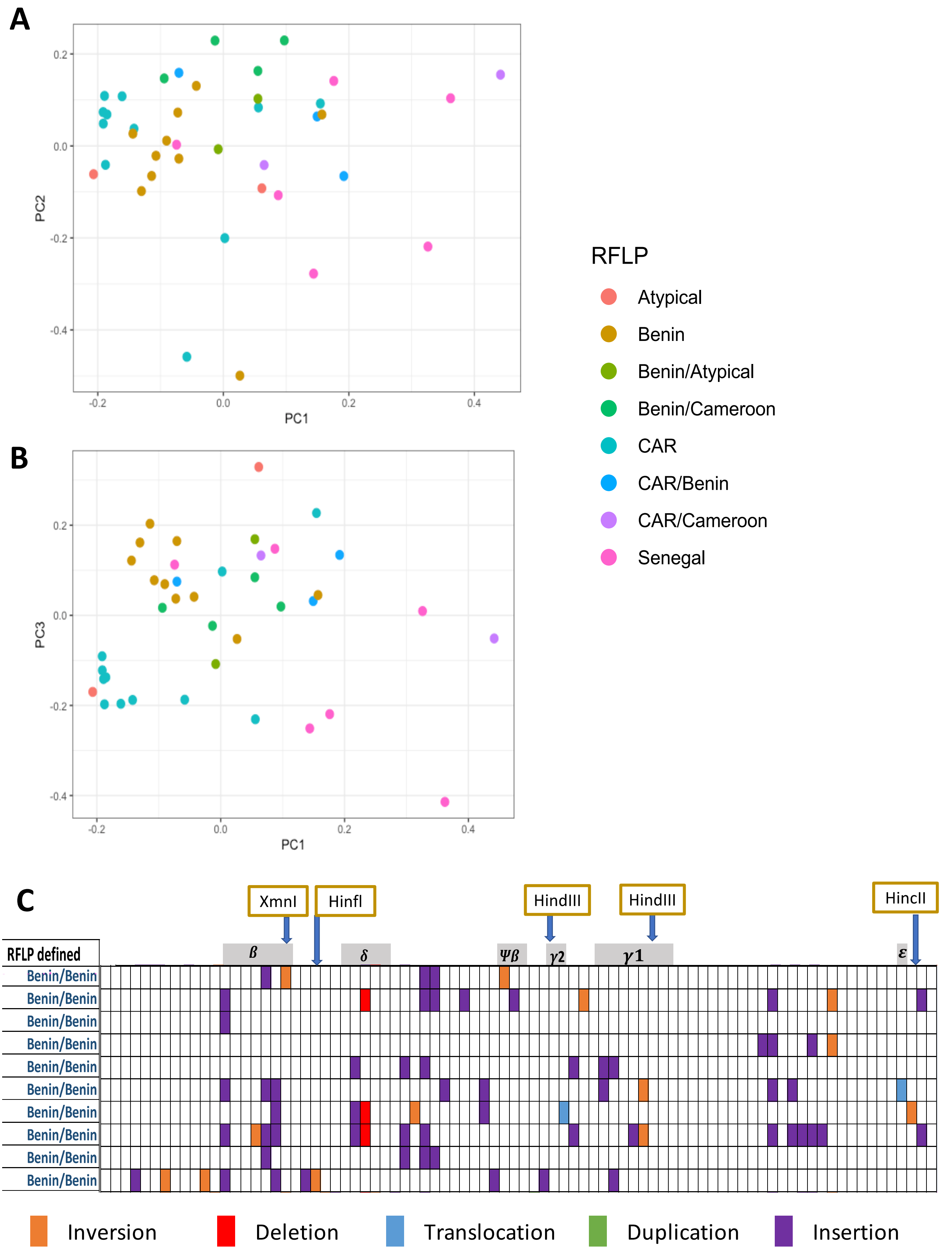

Figure S9. Beta-globin variation on RFLP defined haplotypes. A and **B** show principal components analysis of haplotype combinations by individual using LRS SVs. **C** shows SVs observed in individuals homozygous for the 'Benin' haplotype.

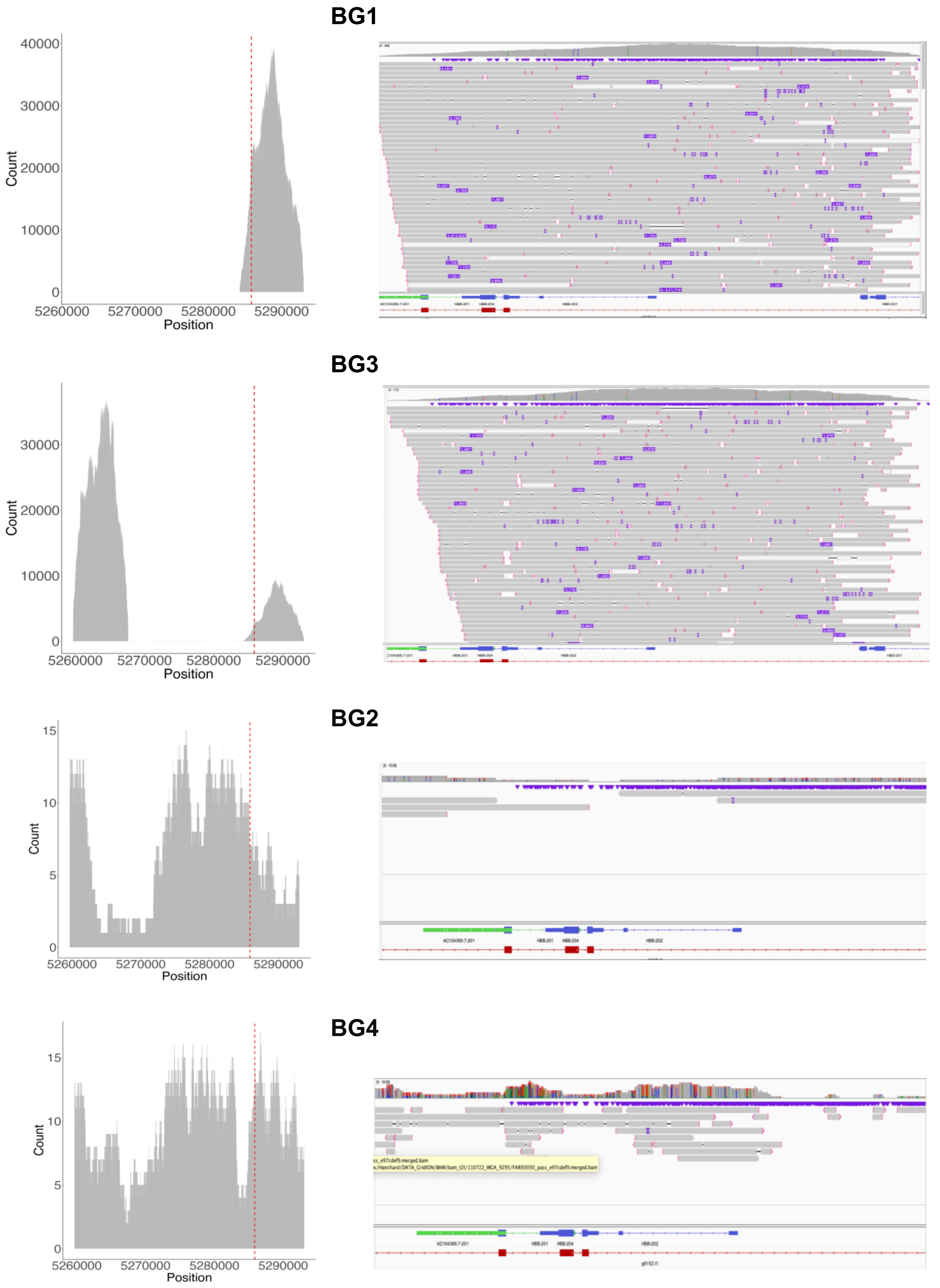

Figure S10. Oxford Nanopore Technology (ONT) validation sequencing (labelled BG1-BG4 in Figure 4). Left panels: Total sequence read coverage across a 30kb stretch on chromosome 11 including at *HBB* (dotted red line). The X-axis shows the genomic position using T2T-CHM13.v2 coordinates. The Y-axis shows the total count of ONT reads. Right panels: IGV visualization screenshots displaying sequencing reads with multiple insertions (purple bars) across *HBB* labelled in basepairs.
