# Supplementary material for: Mapping long-read sequences to a complete reference genome reveals uncaptured variants across the β-Globin cluster in Africans with sickle cell disease
Source: HGG Adv. 2026 Sep 4;7(4):100669. doi: 10.1016/j.xhgg.2026.100669 (PMC13651657; doi:10.1016/j.xhgg.2026.100669)
Supplement: Supplementary file 2 [file mmc3.pdf]

HGG
Advances

ARTICLE

Mapping long-read sequences to a complete reference genome reveals uncaptured variants across the β-Globin cluster in Africans with sickle cell disease

Pamela Lurie,[1,2,3] Medhat Mahmoud,[4] Aparna Haldipur,[3] Yixing Han,[3] Qing Li,[3] Edmond Wonkam Tingang,[3] Natalie Asmus,[3] Emilyn Banfield,[3] Nancy J. Hall,[1,2] Bamidele Tayo,[5] Richard Cooper,[5,16] Titilola Akingbola,[6] George Ayodo,[7] Stephen Obaro,[8,9] Guillaume Lettre,[10] Qing Meng,[4] Shalini Jhangiani,[4] R. Alan Harris,[1,4] Kim C. Worley,[1,4] Juyhun Kim,[11,12] Arang Rhie,[11] Adam M. Phillippy,[11] Fritz Sedlazeck,[1,4,13] Ambroise Wonkam,[14,15] and Neil A. Hanchard[1,2,3,17,*]

Summary

African genomes are marked by extensive complexity in the number and distribution of variants yet remain under-represented in genetic databases and the human reference genome. This gap in representation limits the broad application of genomic medicine. Sickle cell disease (SCD)—one of the most common monogenic diseases—has its highest prevalence in Africa, and variation in disease severity has consistently been linked to the β-globin locus, including levels of fetal hemoglobin (HbF). Modulation of HbF is central to current SCD gene therapies; however, the inherent complexity and variation at the locus in African genomes present a challenge to translating these advances to Africa. Here, we align single-molecule long-read sequencing (LRS) targeted to the β-globin region to the human genome build 38 (hg38) and telomere-to-telomere (T2T)-CHM13.v2 genome references in 40 individuals with SCD, predominantly recruited from three African countries. We demonstrate that the expanded T2T-CHM13.v2 reference sequence at this locus reduces structural variant (SV) calls by 70% and uncovers uncaptured single-nucleotide variants (SNVs). Across the cluster, we report 343 SVs and 196 SNVs that have not been previously reported, including in LRS data from the All of Us project. By including African populations from ethnolinguistic groups that have not been previously surveyed, we improve variant resolution and bolster evidence for observed variation. Finally, we identify a common ∼4 kb insertion locus overlapping the *HBB* promoter among individuals with high HbF. These results demonstrate the utility of combining a comprehensive reference genome with LRS in African populations to uncover genomic variation at disease-associated loci.

Introduction

As the ancestral home of modern humans, populations from continental Africa are known to have more genetic variation than individuals from other continental groups,[1,2] and, as a consequence of a deeper ancestral tree, multiple migration events, and broad ecological variation,[3] they harbor substantial and complex intra-continental genetic variation as well.[4–6] Much of this genetic variation is not captured in modern large-scale genetic databases,[7] and this is reflected among genome-wide association studies (GWASs).[8–10] Added to this genomic data gap are studies suggesting that the commonly utilized human genome reference sequence (human genome build 38 [hg38]) is lacking substantial stretches of DNA found among persons of predominant African ancestries.[11] The lack of African data means that the full extent of human genetic variation remains uncertain, limiting the equitable application of genomic medicine to precision medicine agendas (e.g., rare variant interpretation[7] or application of polygenic risk scores[10,12–14]).

This knowledge gap has implications for addressing genomic disorders that are commonly seen among populations of diverse ancestries. One such example is sickle cell disease (SCD; OMIM: 603903), which includes a group of recessive Mendelian disorders characterized by the inheritance of two abnormal copies of *β-globin* (*HBB*), resulting in the formation of an abnormal adult β-hemoglobin

[1]Department of Molecular and Human Genetics, Baylor College of Medicine, Houston, TX, USA; [2]USDA/ARS Children's Nutrition Research Center, Baylor College of Medicine, Houston, TX, USA; [3]Childhood Complex Disease Genomics Section, Center for Precision Health Research, National Human Genome Research Institute, National Institutes of Health, Bethesda, MD, USA; [4]Human Genome Sequencing Center, Baylor College of Medicine, Houston, TX, USA; [5]Department of Public Health Sciences, Parkinson School of Health Sciences and Public Health, Loyola University Chicago, Maywood, IL, USA; [6]Department of Hematology, University of Ibadan, College of Medicine, University College Hospital, Ibadan, Nigeria; [7]School of Health Sciences, Jaramogi Oginga Odinga University of Science and Technology, Bondo, Kenya; [8]International Foundation Against Infectious Disease in Nigeria, Gwarimpa Abuja, Nigeria; [9]Department of Pediatrics, University of Alabama at Birmingham, Birmingham, AL, USA; [10]Montreal Heart Institute and Department of Medicine, Faculty of Medicine, Université de Montreal, Montreal, QC, Canada; [11]Center for Genomics and Data Science Research, National Human Genome Research Institute, National Institutes of Health, Bethesda, MD, USA; [12]Department of Biomedical Sciences, Seoul National University College of Medicine, Seoul, Republic of Korea; [13]Department of Computer Science, Rice University, 6100 Main Street, Houston, TX, USA; [14]McKusick-Nathans Institute & Department of Genetic Medicine, Johns Hopkins University School of Medicine, Baltimore, MD 21205, USA; [15]Division of Human Genetics, University of Cape Town, Cape Town, South Africa
[16]Deceased
[17]Lead contact
*Correspondence: neil.hanchard@nih.gov
https://doi.org/10.1016/j.xhgg.2026.100669.
Published by Elsevier Inc. on behalf of American Society of Human Genetics.
This is an open access article under the CC BY license (http://creativecommons.org/licenses/by/4.0/).

protein (hemoglobin S [HbS]). Globally, 300,000 babies with SCD are born each year, with more than 70% of these in Africa.[15,16] Close to half of the world's SCD population lives in Nigeria, where the rate and cost of hospitalization for children with SCD present a significant financial burden.[17]

HBB is located within a ~60 kb β-globin cluster on chromosome 11p15.5 in tandem with other developmentally expressed β-globin genes that are responsible for the production of β-hemoglobin both during and after fetal development. The β-globin cluster includes the two gamma-globin genes (*HBG1* and *HBG2*) that are responsible for the production of fetal hemoglobin (HbF),[18] which stabilizes sickled hemoglobin and thus reduces the overall severity of SCD.[19,20] GWASs have identified several loci associated with HbF production,[21,22] including signals within the β-globin cluster, although specific causal variants have remained difficult to pinpoint. For example, a well-known polymorphism characterized by an *Xmn1* restriction site (*rs7482144*) upstream of *HBG2* is consistently associated with HbF levels,[23] but the variance attributable to this site is highly inconsistent across populations and appears to depend on the underlying genetic background.[23,24] More recent work has demonstrated that the developmentally timed expression of β-hemoglobin is regulated through complex interactions between transcription factors acting in *cis* across the β-globin cluster in tandem with local genomic enhancers, including the upstream locus control region (LCR).[25,26] These observations, combined with recent advances in gene editing technologies, have further centered attention on manipulating genes and genetic variation within the β-globin cluster for therapeutic benefit.[27,28] Despite these advances and pride of place of SCD as the "first molecular disease,"[29,30] natural genetic variation in and around the β-globin cluster among Africans remains unmapped, and genomic studies of *HBB* in Africans are rare, portending a potential therapeutic gap to follow the genomic one.

In part, the relative lack of clarity at the locus reflects the underlying genomic complexity, which includes reportedly extensive and complex structural rearrangements,[31,32] difficult-to-sequence repetitive regions in the β-globin cluster, and a recombination hotspot upstream of the *HBB* promoter.[33,34] These genomic features exist within a context of strong signatures of selection on HbS[35] that vary with continental geography.[36] As a result, understanding the genomic variation across the β-globin cluster is particularly important for populations of African ancestries, who bear the greatest burden of SCD, and could thus benefit the most from forthcoming therapeutic interventions, but who also harbor the most and the most complex genetic variation.

Single-molecule long-read sequencing (LRS) offers the ability to sequence the intact native DNA strand, thereby facilitating the identification of structural and copy-number variation, including complex rearrangements that might be "hidden" from other genome-scale sequencing technologies.[37] LRS also improves sequencing across complex genomic regions, allowing for the resolution of highly diverse or repetitive regions. This technology was central to the development of the complete (telomere-to-telomere [T2T-CHM13]) human genome,[38] which has since been incorporated as an alternate human genome reference that includes the 8% of missing heterochromatin DNA and >200 Mb of DNA sequence relative to hg38.

Here, we apply LRS across the β-globin cluster in African- and African-diaspora individuals with SCD to provide a comprehensive exposition of the underlying genetic variation. We align LRS reads to both the GRCh38 and T2T-CHM13.v2 genome references and characterize previously undescribed structural variants (SVs) and single-nucleotide variants (SNVs) across groups. We then illustrate the potential gains of updated mapping by seeking clinically relevant SVs with *cis*-regulatory potential (Figure S1).

Subjects and methods

Sample collection

We relied on clinically convenient, de-identified samples representative of our target population (African participants with SCD), some of which were obtained as part of previous studies. African samples were available from four sites in three countries (Figure 1; Table S1):

- From Kenyan (Luo) individuals >5 years old receiving care at the Lwak Hospital in western Kenya. DNA was obtained from whole-blood samples procured after permission for genetic studies of SCD was granted by the Ethics Board of Maseno University, Kenya.
- From Cameroon individuals aged 5 years and older recruited from the Yaoundé Central Hospital and Laquintinie Hospital in Douala, Cameroon as part of a study of HbF approved by the National Ethical Committee Ministry of Public Health, Republic of Cameroon (no. 033/CNE/DNM/07), and the University of Cape Town, Faculty of Health Sciences Human Research Ethics Committee (HREC RE: 132/2010). DNA was extracted from whole-blood samples, and HbF was measured at the University of Cape Town in participants who had not received a blood transfusion or been hospitalized in the previous 6 weeks. No patients were being treated with hydroxyurea.
- From Nigerian individuals >12 years old identifying as being from the Yoruba ethnolinguistic group, recruited at the Clinic at the University College Hospital in Ibadan. DNA was obtained from whole-blood samples and shipped to Loyola University in Maywood, IL, USA, as part of a study of HBF genetics in painful crises of SCD. Recruited individuals did not have acute vaso-occlusive pain and were at least 4 weeks removed from blood transfusion or a vaso-occlusive crisis requiring medical attention.[39,40]
- From Nigerian individuals with SCD < 5 years old identifying as being from the Hausa ethnolinguistic group presenting clinically with acute febrile illness, pneumonia, or meningitis to **Murtala Specialist Hospital Kano, University of Abuja Teaching Hospital in Gwagwalada, Nigeria**. DNA was extracted from blood samples taken in accordance

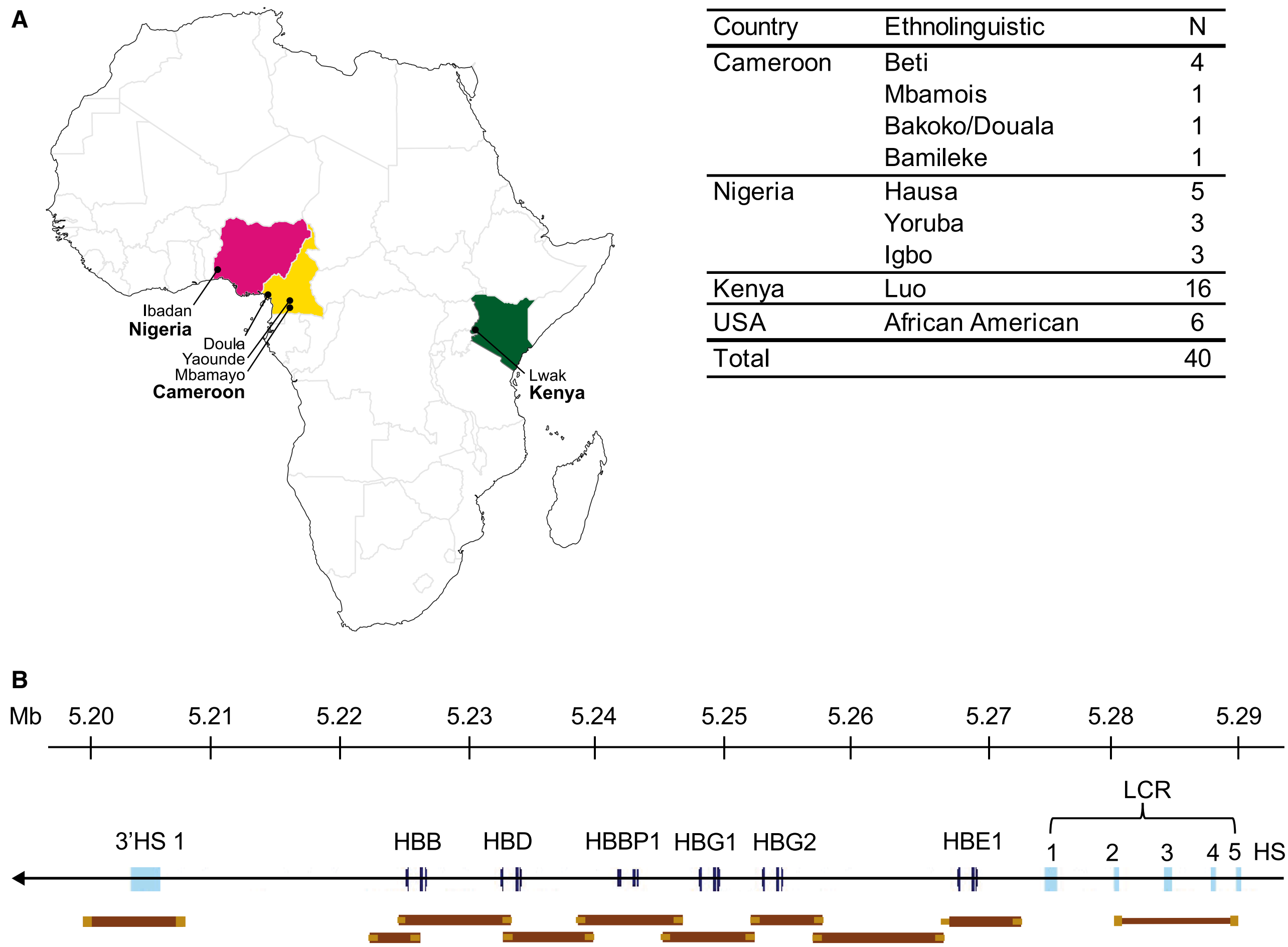

Country	Ethnolinguistic	N
Cameroon	Beti	4
	Mbamois	1
	Bakoko/Douala	1
	Bamileke	1
Nigeria	Hausa	5
	Yoruba	3
	Igbo	3
Kenya	Luo	16
USA	African American	6
Total		40

Figure 1. β-Globin locus amplification in Africans with SCD
(A) Participant representation among African regions, cities, and ethnolinguistic groups.
(B) Amplicon tiling across the major coding genes, 5′ and 3′ hypersensitive sites (HSs), and locus control region (LCR) in the β-globin cluster (amplicon sizes are represented by the brown bars with the gold ends illustrating flanking primers).

with the stipulations of the University of Abuja Teaching Hospital, Kano State Health Services Management Board.[39,40]

At each institution, HbF levels were measured using locally available high-performance liquid chromatography (HPLC). All participants had confirmed homozygous S disease (HbSS), and informed consent (self or parent/guardian) was obtained from all individuals to use their samples for genetic studies related to SCD but was not specific to this study.

RFLP haplotype analysis

To characterize classical sickle haplotypes, restriction fragment-length polymorphisms (RFLPs) across the β-globin cluster were genotyped in 145 blood and buccal DNA samples according to published protocols[41] (summarized in Table S2 and supplemental methods). One-hundred seventeen (117) samples had cleavage patterns that were consistent with one of the classical sickle haplotypes; 14 (12%) were heterozygous, and the remaining 103 were homozygous (Table S1). Samples were further prioritized on total available volume and quantity of DNA, as well as sickle haplotype (Benin, Cameroon, or Senegal) to identify a set of 34 samples (23 homozygotes and 11 heterozygotes) for LRS. Additionally, three homozygous Senegal and three heterozygous Benin/Cameroon samples from the USA were available in-house through previously approved studies[42] and were included to improve representation across all four haplotypes. A final total of 40 samples consisting of 27 Bantu/CAR, 25 Benin, 12 Senegal, seven (7) Cameroon, and four (4) atypical haplotypes were made available for LRS.

Amplicon tiling

To capture variation within and outside of the β-globin cluster, long-range PCR was performed using the Primestar GXL long-range PCR protocol (Takara USA Biosciences) across eleven (11) amplicon regions (Table S3), each approximately 10 kb in size. Nine amplicons spanned the β-globin cluster (71 kb), while the other two amplicons flanked the cluster ~14.5 kb upstream of the adult β-globin gene (*HBB*) and ~300 kbp downstream of the LCR (Figure 1). Amplicons were generated using independent barcoded primers for each of the 40 selected samples (Table S4). The resulting amplicons were cleaned using ExoSAP (Thermo Fisher Scientific) according to the manufacturer's recommendations and pooled for sequencing.

SMRT-cell LRS

Pooled amplicons were subjected to long-read single-molecule real-time (SMRT) technology (Pacific Biosciences [PacBio], San Diego) sequencing. Amplicon pools were end repaired and ligated

with SMRT adaptors from the SMRTbell Template Prep Kit 1.0. Final libraries were loaded with the v.2.1 sequencing kit on a single Sequel I SMRTcell at a concentration of 4–8 pM. The sequencing data were collected for 10 h on the sequencer and analyzed using SMRTLink 5.0.1 software. A range of 7.1–13.0 Gb of raw data were generated for each amplicon pool with a read-length N50 of 2.25–4.25 kb.

LRS mapping

Raw SMRT-cell reads were de-multiplexed using LIMA (web resources), and only reads consistent with the barcode schema were subsequently used. PRINCESS v.1.0[43] with the "phase" option and preset parameters "princess phase -d output directory -r clr -f GRCh38.fa -a ngmlr" was used to identify and phase SVs and SNVs. PRINCESS uses Minimap2 v.2.17,[44] Sniffles v.2,[45] and Clair 3[46] with the adequate model and phases the data using WhatsHap v.0.18.[47] We also aligned the reads to the chm13 reference (T2T)[38] using PRINCESS v.1.0[43] with the same preset parameters "princess phase -d output directory -r clr -f chm13v2.0.fa" with the "phase" command to call and phase SNVs and SVs.

LRS quality review

The per-nucleotide coverage of the hg38 reference human genome was calculated for each sample and amplicon using SAMtools v.1.11[48] (web resources). To determine the minimum read-depth threshold to apply during filtering, we calculated the median, 25th, and 75th percentiles for coverage across all samples (Table S5). Gnuplot v.5.2 (web resources) was used to plot the read-length distribution. The depth of coverage for each sample varied by amplicon (Figure S2), although the mean coverage for all samples across the β-globin cluster was ~1,600×. The minimum N50 value (50% of sequenced base pairs) across all amplicons was 8,905 bp (Figures S3 and S4).

T2T-CHM13.v2 annotation

To annotate variants in ANNOVAR based on the T2T-CHM13.v2 reference, we used several databases (Table S6) that included SNP information from dbSNP, variants associated with phenotypes such as GWASs, and ClinVar SNPs, among others. We also downloaded exclusive features of the T2T-CHM13.v2 reference (e.g., 1000 Genomes Project allele frequencies by population, non-syntenic regions compared to GRCh38, resolved regions that had issues in GRCh38 [such as gaps], and more comprehensive annotations of centromeres/satellite repeats in CHM13).[49] For databases based on GRCh38 reference coordinates, we converted coordinates to T2T-CHM13.v2 using CrossMap[50] with grch38-chm13v2.chain.[49] All files are available to download from the CHM13 GitHub page (web resources).

The datasets were then converted to a format acceptable by ANNOVAR.[51] The ANNOVAR program employs three specific annotation methods tailored to database features, requiring databases to be in formats that match each mode. For gene-based annotation, GFF3 files were converted to GTF and GenePred formats using the UCSC tool *gtfToGenePred*. This mode also necessitated a transcript FASTA file, obtained using retrieve_seq_from_fasta.pl, an accessory program of ANNOVAR, to retrieve genomic nucleotide sequences from cDNA annotations.

The second mode of ANNOVAR annotation is region-based annotation. We downloaded the GWAS Catalog[52] association summary file (v1.0-associations_e110_r2023-12-07; web resources) and formatted it to match that of the ANNOVAR GWAS database to ensure compatibility with ANNOVAR. Other BED format region files, such as cenSat or accessibility regions, can then be used without requiring specific transformation.

Lastly, filter-based annotation can be divided into two categories: allele frequency and others. For 1000 Genomes Project allele frequencies, VCF files were transformed into the same format as an ANNOVAR 1000 Genomes Project allele frequency database by population. Additionally, other databases, including dbSNP and ClinVar, were transformed into formats corresponding to each database in ANNOVAR. Occasionally, allele frequencies were extremely different between populations, particularly in ClinVar; this resulted in the major or reference alleles being flipped, challenging annotation. In such cases, we used GATK (v.4.5.0.0) LiftoverVcf with the flag RECOVER_SWAPPED_REF_ALT = true based on the CHM13v2.0 reference to check and correct allele flips simultaneously.

SVs

A total of 397 SVs were called using Sniffles v.2.[45] Previously unreported SVs were defined as variants without overlapping breakpoints within 100 bp of the same type of structural event in the Database of Genomic Variants (DGV)[53] and gnomAD (v.3.1) (web resources). Additional filtering removed 32 translocations and 22 variants that were located outside of the β-globin locus thought to be data artifacts. SURVIVOR was used to merge SVs with breakpoints within 1 kb and resulted in 182 merged SVs, of which 14 events within the region were observed in ≥4 samples.

SNVs

A total of 13,581 SNVs were identified using Clair 3[46] with T2T-CHM13.v2 as the genome reference. ANNOVAR was used to query, flag, and then remove SNVs seen in the ExAC, dbSNP, avSNP, ESP, ClinVar, gnomAD, and 1000 Genomes databases; the remaining 1,536 SNVs were described as uncaptured. SNVs with an allele fraction <0.3 and/or read depth <30× were removed, leaving 196 uncaptured SNVs. ANNOVAR was also used to functionally annotate uncaptured SNVs. The same approach was taken to filter SNVs called against the hg38 reference.

Circular plots

Circular plots were constructed using the BioCircos[54] package in R (see data analysis) using coordinates of variants, sequenced regions, and gene coordinates retrieved from the UCSC genome browser. Comparisons between T2T-CHM13.v2 and hg38 coordinates were manually recorded from NCBI genome data viewer assembly alignment and assembly difference graph tracks (for shared coordinates) comparing T2T-CHM13.v2 to the GRCh38.p14 alignment track.

Data analysis

Primary analyses and data curation were performed using R 4.1.3 GUI 1.77 High Sierra build (8051) and RStudio 2022.02.0+443 "Prairie Trillium" for macOS.

Orthogonal LRS validation using ONT

We attempted to validate the insertions observed across *HBB* in four samples (BG1, BG2, BG3, and BG4). DNA samples from these individuals were initially purified using phenol-chloroform. Due to the limited quantity of remaining DNA,

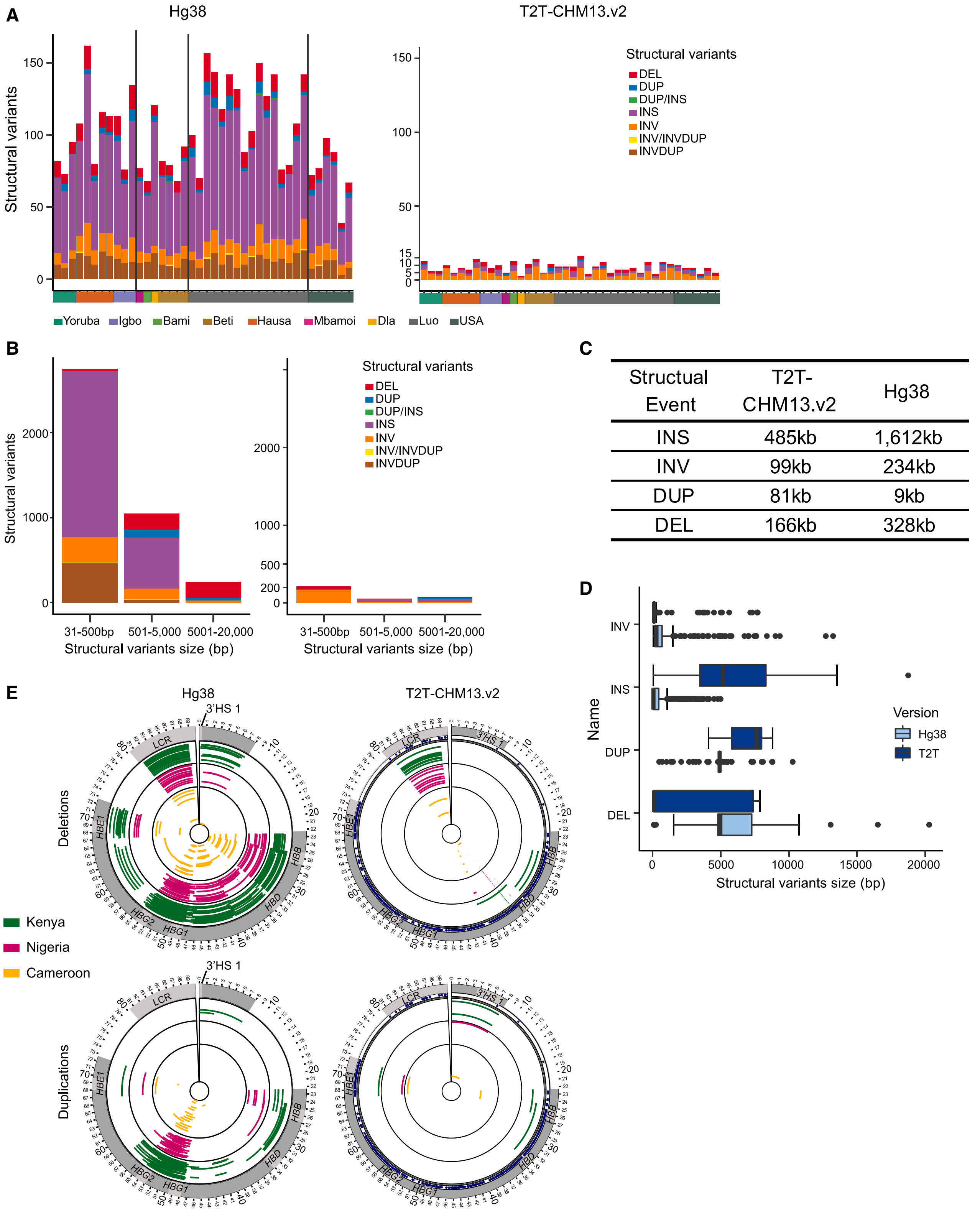

Structual Event	T2T-CHM13.v2	Hg38
INS	485kb	1,612kb
INV	99kb	234kb
DUP	81kb	9kb
DEL	166kb	328kb

Figure 2. Structural variant calling in the β-globin region

(A) Number and classes of unreported structural variants called with hg38 and T2T-CHM13.v2 references in each individual. Samples are grouped by geographical location; from left to right: Nigeria, Cameroon, Kenya, and USA.

(B) Distribution of structural variant sizes using hg38 (left) and T2T-CHM13.v2 (right).

(C) Base-pair differences between T2T-CHM13.v2 and hg38 for structural variant types.

(D) Boxplots of SV lengths called by hg38 and T2T-CHM13.v2. Boxplot shows median (bold line); upper and lower quartile (box), and maximum and minium range for the data, with outliers show as individual dots.

(legend continued on next page)

whole-genome amplification was performed, followed by library preparation and sequencing according to published protocols from Oxford Nanopore Technologies (ONT; Oxford, UK; web resources). These protocols were further optimized to include an Ampure bead clean-up instead of a custom buffer clean-up step during whole-genome amplification, which resulted in a notable enhancement of library yield. Samples were sequenced on a GridION X5 Mk1 (GXB03474) Sequencer, programmed for a 72-h run in adaptive sampling mode, with chromosome 11 as the reference for enrichment. The 72-h sequencing run involved three consecutive library loads onto the R9.4.1 FLO-MIN106D flow cell, each temporarily halted at 24-h intervals for a flow cell wash using the Flow Cell Wash Kit XL (EXP-WSH004-XL), followed by priming and reloading with a fresh 75 μL of the library preparation. The inclusion of these optimization steps resulted in 102.39 GB of data and 3.52 million reads for BG1, 191.16 GB of data and 6.41 million reads for BG2, 283.37 GB of data and 11.25 million reads for BG3, and 352.7 GB of data and 14.62 million reads for BG4. The N50 for the four samples ranged from 2.81 to 4.93 kb.

Base calling of raw GridION sequencing data was done using Guppy v.6.5.7 with the high-accuracy model (dna_r9.4.1_450bps_hac.cfg). The resulting base-called reads were then mapped to the human reference genome T2T-CHM13.v2 using Minimap2[44] (v.2.24-r1122). Aligned reads were merged and sorted using SAMtools v.1.19, and the resulting alignment files were stored in BAM format. SVs were called using Sniffles v.2.3.2. A combination of SAMtools and a custom script was employed for the selection of reads containing large insertions. All software was employed using default parameters. Sequencing reads were visualized using IGV v.2.16.0. SAMtools depth was utilized to determine the number of nucleotides aligned to the targeted region. R v.4.3.1 was used for plotting the depth of the targeted region.

β-Globin loci SVs from All of Us project LRS data

Within the All of Us (AoU) Research Program Control Tier v.8, 2,800 individuals have both short-read whole-genome sequencing (srWGS) and LRS data available; LRS data are grouped by sequencing facility and platform into 11 cohorts. Based on genetic ancestry inferred from srWGS data, 1,250 individuals of African ancestry with LRS data were selected. These samples are distributed across three processing cohorts: 959 from Control Tier v.7 callsets; 253 from the BI (Broad Institute) processing center and 38 from the BCM (Baylor College of Medicine) processing center within Control Tier v.8. The minimum coverage across these cohorts ranged from 12× to 25× (Table S7). Single-sample SV files generated by Sniffles2 were merged into multi-sample VCF files within each processing cohort using Sniffles2. Prior to comparison, we retained only PASS SV calls >30 bp within the T2T-CHM13.v2 β-globin interval chr11:5259000–5350000 and excluded translocations, breakend (BND) calls, low-confidence or non-PASS calls, and calls outside this interval. These filtered AoU SVs were compared with SVs identified in our study cohort, which were merged across the 40 study individuals using SURVIVOR.

Results

We started by evaluating classical sickle RFLP haplotypes (subjects and methods) in 234 individuals with SCD from Nigeria ($n = 93$), Cameroon ($n = 27$), Kenya ($n = 97$), and southwest USA ($n = 17$) (Table S1). From this group, we chose 34 individuals from continental Africa to represent each of the four sickle haplotypes that have been commonly described—*Senegal*, *Cameroon*, *Central Africa*, and *Benin* types (Figure 1)—and added three *Senegal* and three heterozygous *Benin/Cameroon* sickle haplotype samples from southwest Louisiana[42] to improve the representation of these haplotypes. We then chose to undertake a tiled amplicon strategy across the region (Figure 1)—in part, this represented cost constraints at the time of undertaking the study, as well as practical considerations of pooling limited and precious DNA samples while also ensuring an adequate number of reads from SMRT-cell sequencing. For each of the 40 individuals, an 82 kb region in and around the β-globin cluster was amplified using a long-range PCR strategy that included partially overlapping tiled and barcoded primers (Figure 1; subjects and methods). Each amplicon then underwent LRS in a single SMRT cell of PacBio Sequel I. The resulting data were processed for quality control (QC) and aligned to a reference genome before calling SVs, SNVs, and small insertion or deletion (indel) events (subjects and methods; Figure S1).

The T2T-CHM13.v2 reference genome improves SV and SNV calling in the β-globin region

Initially, we aligned QC reads from the 80 assayed African chromosomes to hg38 and used Sniffles[45] to call SVs (subjects and methods). This analysis identified a total of 4,876 SV events (>30 bp), including 2,555 insertions (63%), 502 inversion/duplications (12%), 437 inversions (11%), 409 deletions (10%), 129 duplications (3%), and seven complex events (inversion/inversion duplications [$n = 7$] and duplication/insertions [$n = 3$]) (Figure 2A). The majority of called insertions were small (between 30 and 500 bp), with larger deletion and duplication events (5–20 kb) (Figure 2B), and almost all events (99%) were unique to our dataset, not being observed in either the DGV (v.107)[53] or the genome aggregation database (gnomAD v.3.1).[55] We surmised that some of the 245 kb (total) recurrent and overlapping insertions might represent either uncaptured DNA reference sequences in our African SCD cohort or technological artifacts of the Sequel I platform. To evaluate this, we realigned the LRS raw data to the T2T-CHM13.v2 reference,[38] re-called SVs using the exact same variant-calling pipeline, and compared the number of SV calls across the same 82 kb amplicon region. Relative to hg38, realignment to T2T-CHM13.v2 resulted

(E) Genomic context of identified structural variants. Lighter gray shaded regions represent gene and hypersensitive sites. Blue dots beneath the gene name and sequenced region track on T2T-CHM13.V2 circular plots represent areas of non-alignment between the hg38 and T2T-CHM13.v2 reference genomes. Note: the location of the 3′HS-1 site is further downstream of *HBB* in T2T-CHM13.v2. INS, insertion; DEL, deletion; DUP, duplication; Inv, inversion.

in a reduction in all classes of SV calls, with 93% fewer SVs (4,876 vs. 343 uncaptured SVs [>30 bp in length]; Figure 2A). The largest reduction was in the number of small (>30 bp length) insertions (2,555 vs. 83 insertions; 97%), with other classes being reduced by ~60%–90% (437 vs. 180 inversions [59%]; 409 vs. 68 deletions [84%]; and 129 vs. 12 duplications [91%]; Figure 2B). The same trend was observed with translocations (644 hg38 calls vs. 32 T2T-CHM13.v2 calls), although these occurred in the sequenced region outside of the β-globin locus (3′ hypersensitive site [HS]-1) and were not included in downstream analyses. Notably, there were no complex events (inversion/inversion duplications, inversion/duplications, or duplication/insertions) called from the T2T-CHM13.v2 alignment. Overall, alignment to the T2T-CHM13.v2 reference resulted in fewer total SV base pairs (1,126,800 bp among insertions, 162,379 bp among deletions, 134,613 bp among inversions, and 15,535 bp among duplications) relative to hg38 (Figure 2C), and, except for insertions, variants called with T2T-CHM13.v2 had less size variability compared to hg38 variant calls (Figure 2D).

We considered that the noise reduction of the T2T-CHM13.v2 alignment might primarily reflect the resolution of difficult-to-sequence regions. To explore this further, we plotted differences between T2T-CHM13.v2 and hg38 alignments across the β-globin locus, alongside the SVs called using both alignments. We observed that 67 of the T2T-CHM13.v2-resolved gaps across the β-globin locus contained 1,114 kb of hg38-called SVs. The improved resolution afforded by this additional sequence was evident across all variant classes evaluated (Figure S5) and had striking effects at specific loci. For instance, alignment to hg38 resulted in large duplication calls across the gamma-globin genes *HBG1* and *HBG2*, which share approximately 96% sequence identity; these duplications were not called with alignment to T2T-CHM13.v2 (Figure 2E). Interestingly, although most deletion calls were resolved after realignment, 40% of the individuals sequenced (16/40 samples) still had deletion calls spanning 8–10 kb across the LCR (Figure 2E), placing the expected LCR sequence at different locations across individuals.

Although realignment to T2T-CHM13.v2 resulted in a reduced number of SV events, the additional DNA reference sequence resulted in an *increased* number of SNV calls relative to hg38 (Figure S6A). A total of 196 previously uncaptured SNVs at 188 unique positions were observed with alignment to T2T-CHM13.v2 (Table S8), representing 78% more unique calls than aligning to hg38 (114 total SNVs at 41 unique positions). The site frequency spectrum of T2T-CHM13.v2-called SNVs was also more consistent with variant discovery, with the vast majority being singletons (Figure S6B). This latter group included five non-synonymous missense changes not observed using hg38, of which one was predicted to be damaging (pathogenic) to *HBB*. As with many of the SVs observed, these SNVs were not observed in any of the public genome databases we interrogated (ExAC, 1000 Genomes, dbSNP, and ClinVar; see subjects and methods) and were thus considered unique to this study's samples. Five SNVs were observed in more than one sample and were found to be either intergenic (two between *HBG1* and *HBG2* and one between *HBG2* and *HBE1*) or intronic (one in *HBG1* and one in *HBE1*) (Figure S6C); the latter variant was annotated to the *HBE1* promoter in hg38 but within the *HBE1* coding region in the T2T-CHM13.v2 reference genome.

Genomic variation at the β-globin locus differs by African SCD population

Although the number of individuals evaluated per population was small—limiting the power of direct comparisons—we did note that individuals with SCD from Kenya (all of whom identify with the Luo ethnolinguistic group) had the most unreported variation—139 SVs and 63 SNVs—among the geographical groups assessed. Because this group also constituted our largest sample, we undertook an exploratory comparison of this contribution by randomly down-sampling the Luo dataset to match the smallest geographical sample size ($n = 9$); this reduced the total number of SVs but still left 63 SNVs, the majority of which were still observed in this ethnolinguistic group. Samples from Nigeria ($n = 11$) had more total uncaptured variants (99 SVs and 83 SNVs) than samples from nearby Cameroon ($n = 7$; 62 SVs and 19 SNVs), although, consistent with the small sample sizes, the median numbers of SV events across the three African countries were not statistically different (Figure S7). Most of the Nigerian samples were from individuals self-reporting as either Yoruba or Igbo—two populations well represented in the 1000 Genomes Project and in public databases of short-read sequence data. Although speculative, the number of uncaptured variants detected in these groups might reflect the added value of LRS to provide a more unbiased sequence evaluation or could represent variants unique to SCD populations in Nigeria, which has the highest carrier frequency of the HbS allele globally. African Americans with SCD from Louisiana, USA, who have substantial West African ancestry, had the fewest number of total variants ($n = 6$; 43 SVs and 31 SNVs), partially justifying the wider representation of non-admixed African populations.

Classical sickle haplotypes are not reflective of β-globin cluster variation

RFLP-based sickle haplotypes, first described in the 1980s, have been consistently used as proxies for the *cis*-genetic background of the sickle allele and are still used to understand disease modulation in SCD, despite their variable association with SCD severity. Given the improved resolution afforded by LRS, we sought to place the variation observed in the context of RFLP haplotypes by undertaking principal component analysis (PCA) of LRS-observed variation and labeling the samples by RFLP haplotype. In this analysis, we noted that Benin/Benin homozygous and

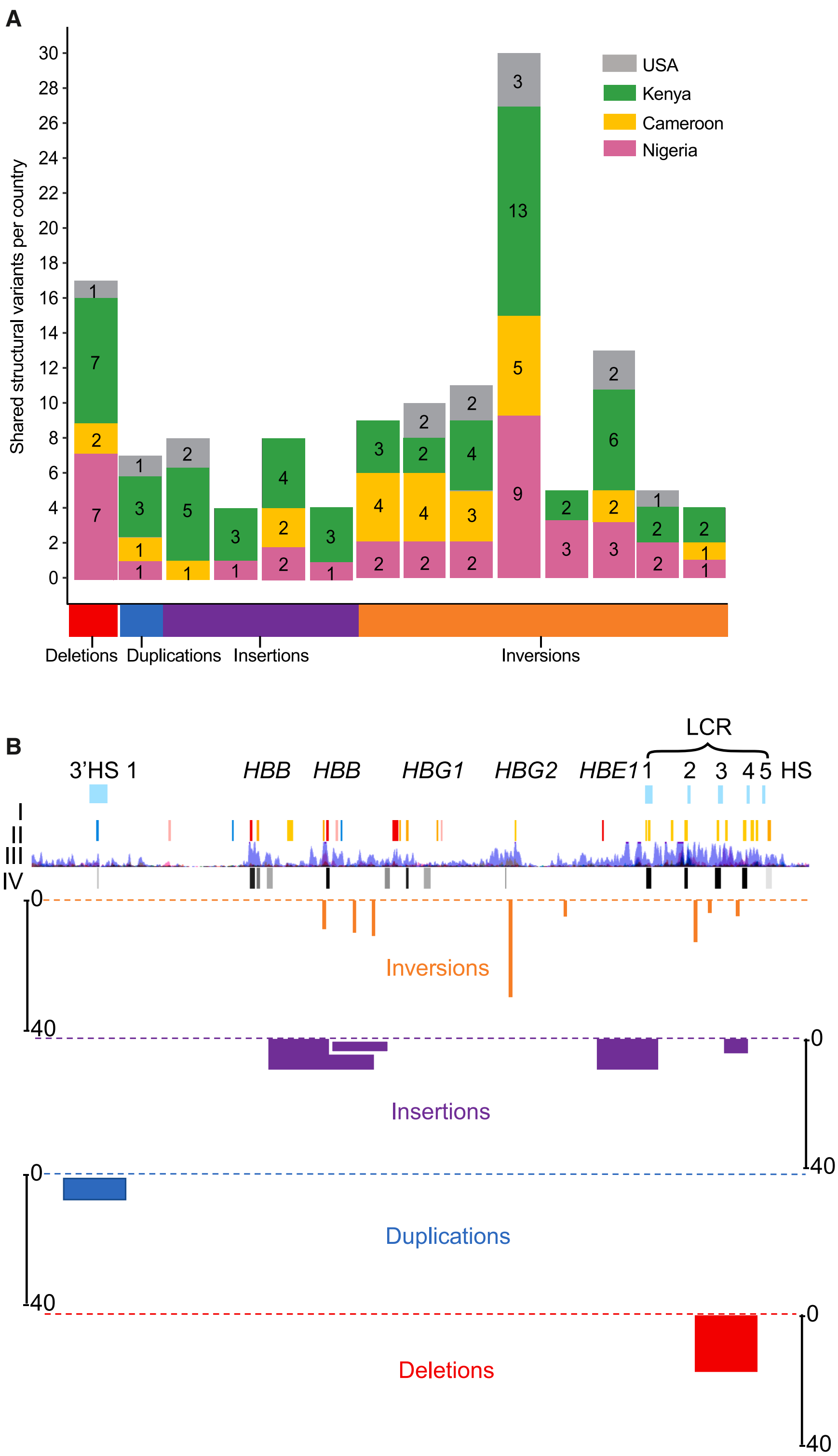

Figure 3. Common structural variants across the β-globin locus in African SCD populations
(A) Bar chart illustrates the number of samples per geographical group with the same structural variant (SV) type. The SV type for each bar is shown on the *x* axis.
(B) Genome locations with the β-globin locus of SVs in (A). Genome track features are given on the *y* axis; I, segmental duplication; II, ENCODE candidate *cis*-regulatory elements (cCREs); III, H3K4Me1sites; and IV, ENCODE transcription factor (TF) chromatin immunoprecipitation sequencing (ChIP-seq) clusters for GATA1. LCR, locus control region; HS, hypersensitivity site.

CAR/CAR homozygous samples clustered closely on PC1 and PC3, although this observation was potentially confounded by geographic distance, as most Benin samples were from Nigeria (West Africa) and most CAR samples were from Kenya (East Africa). There was no distinct clustering of homozygous Senegal, heterozygous, or atypical samples (Figures S9A and S9B). A closer evaluation of SV variation occurring on the background of individuals with Benin/Benin haplotype demonstrated evidence of a new mutation and/or recombination such that few of the common (and presumably older) SVs (e.g., seen in ≥4 individuals) were consistently observed on all or even most haplotype backgrounds (Figure S9C).

Common SVs are observed across the spectrum of Africans with SCD

To reduce the potential for singleton events to have an outsized impact on our downstream comparison, we limited our subsequent analyses to cohort SVs consistently observed in multiple individuals. Given the modest sample size, these SVs are likely to represent common alleles. Because individual SVs can have different breakpoints, we merged variant calls within 1 kb and classified common SVs as those with overlapping genomic regions in four or more (≥4) samples; this resulted in 14 common SVs—one deletion, one duplication, four insertions, and eight inversions (Figure 3A; Table S9). The inclusion of samples from Kenya was important in confirming many of the identified SVs, as they provided additional instances of events that were singleton in other populations. The Luo from Kenya made up 40% of the studied group, yet more than half (15 of 24, 62.5%) of all individuals with insertions were from this group, suggesting that there may be additional "unrepresented references" even beyond that in T2T-CHM13.v2 for these relatively unexplored populations. Further evidence of potential unrepresented DNA sequence in this diverse African population was suggested

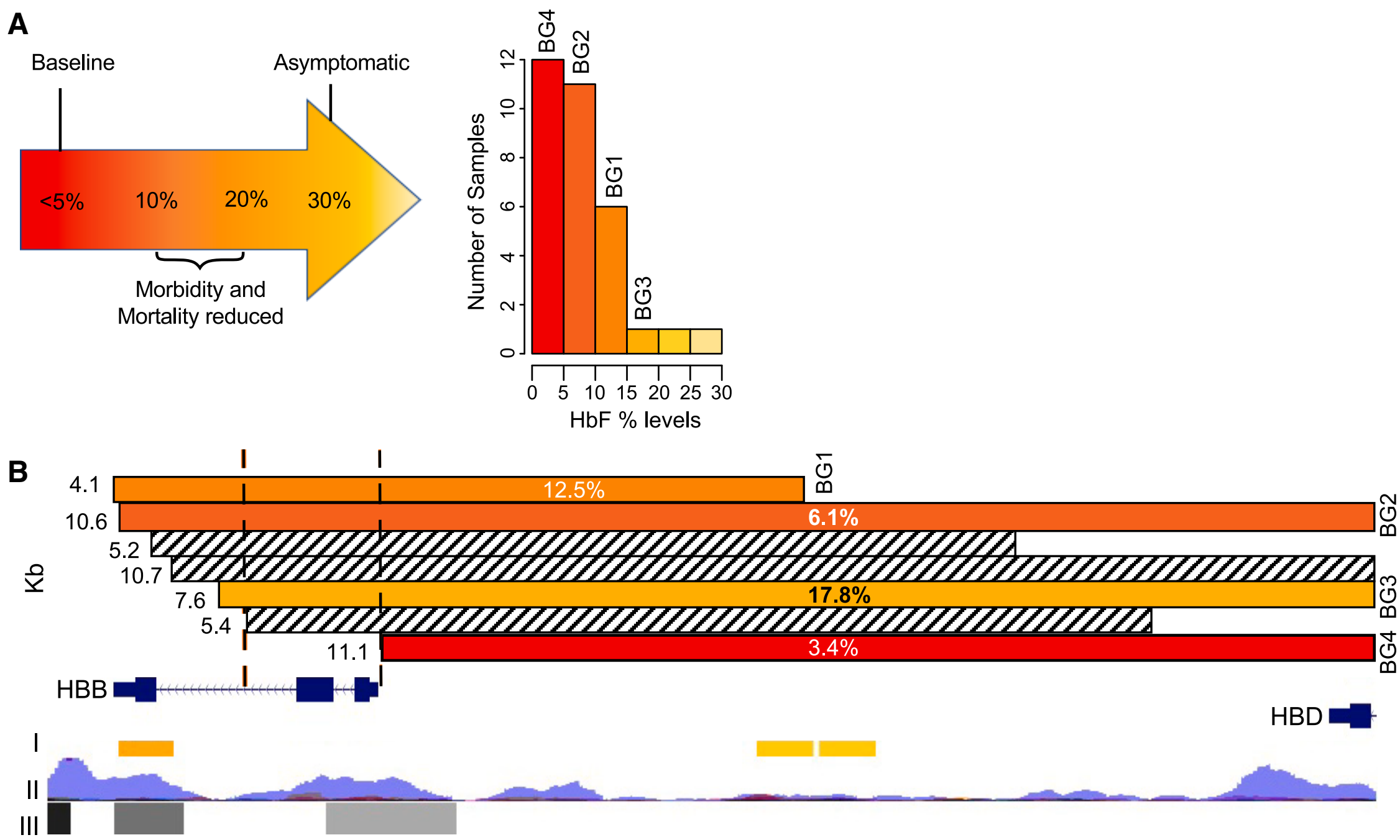

Figure 4. Mapping candidate variants impacting HbF level in African SCD samples
(A) Illustrative correlation between HbF levels and SCD symptoms (left) and available in the recruited African SCD cohort (right). Numbers at each bar represent individuals with common SVs.
(B) HbF candidate insertion overlapping HBB, with the smallest region of overlap (SRO) shown by the dotted lines. The HbF level (when known) is given in the bar, and the length of each SV is given to the left of each bar. Track annotation: I, segmental duplication; II, ENCODE candidate *cis*-regulatory elements (cCREs); and III, H3K4Me1 sites.

by an inversion near *HBG2*, which was observed in 30 of 40 individuals (Figure 3B). Overall, common inversions and insertions were broadly distributed across the β-globin cluster, spanning the exons and regulatory regions of *HBB*, *HBD*, and *HBE1*, while individual, but recurrently observed, duplication and deletion events were observed in the 3′HS and 5′HS regions, respectively.

To provide a broader population context for the occurrence of these SVs, we compared their genomic location, type, and frequency with LRS SV calls from individuals clustering with African-ancestry groups in the AoU project (subjects and methods). Within the β-globin locus interval (chr11:5259000–5350000) on T2T-CHM13.v2, only seven SVs were identified across the more than 1,250 individuals of African ancestry in AoU, with two shared SVs observed in fewer than 4 individuals. None of these SVs fully overlapped the SVs in our study cohort, although four of the seven AoU SVs were within 1 kb of common SVs from the same class in the study cohort (Figure S8). We note that AoU data were generated on different sequencing platforms and utilized differing coverage and merging strategies (subjects and methods; Table S7); as a result, these findings should not be interpreted as a direct quantitative comparison of SV burden. Instead, they provide qualitative population context for the degree of overlap between available African-ancestry LRS callsets and the variants identified in this SCD-enriched African cohort.

LRS-identified variation includes potential disease-modifying candidates

Finally, we sought to illustrate the potential practical implications of an updated reference alignment by attempting to identify potential HbF-modifying SVs in the subset of 32 samples with available HbF levels (Table S1). We started by applying a "forward" filter, in which we focused on T2T-CHM13.v2 SV calls occurring in multiple (≥4) individuals and overlapping ENCODE conserved regulatory elements (CREs) or binding sites known to impact HbF (e.g., regulatory transcription factor GATA1) (Figure 3). Only one SV event met these criteria—a set of seven overlapping insertions in seven individuals, occurring upstream of HBB, varying in size from 4 to 12 kb with breakpoints in or around ENCODE enhancer sites, including a nearby GATA1 transcription factor binding site (Figure 4). The mean HbF level among the five individuals with available HbF measurements was 10% (standard deviation: 6.4%). We then attempted a "reverse" filter approach, focusing first on individuals with HbF levels in the extremes of the distribution, i.e., with either high (≥10%; $n = 9$) or low (≤5%; $n = 12$) HbF, as these cutoffs generally correlate with clinical severity[56] (Figure 4; Table S1). There were no variants exclusive to either group; however, when we considered variants observed in a majority of individuals (>80%; $n = 7$ for high HbF; $n = 10$ for low HbF), the insertion event upstream of HBB identified among individuals with elevated HbF in

our forward filter was the only event meeting the imposed threshold.

Although preliminary, with an insufficient number of events to generate meaningful statistics, we considered the insertion site a potential HbF regulatory candidate in these populations and sought to validate the insertions using an orthogonal LRS platform (ONT) (subjects and methods). We attempted to validate insertions in four samples (labeled BG1, BG2, BG3, and BG4 in Figure 4) for which we had additional DNA. As these DNA samples were limited in quantity and quality, we first performed PCR-free whole-genome amplification before undertaking adaptive sequencing of chromosome 11 on the ONT platform and aligning the resulting reads to the T2T-CHM13.v2 reference. We then applied an *in silico* caller,[45] before manually (visually using Integrated Genome Viewer [IGV]) reviewing putative SV calls within 1 kb of our initial SV for any heterozygous (seen in ~30%–50% of reads) SV calls of a similar class (i.e., insertion, deletion, etc.). Two samples—BG2 and BG4—had insufficient coverage over the region and were not evaluated. In the remaining two samples, we observed reads harboring the same SV class (insertion) and size (4–7 kb) at either the same location (chr11:5285000–5293000) or within >1 kb; however, these calls were only evident in a small subset of the total reads at the site of interest (217 for BG1 and 167 for BG3 out of >30,650 reads) (Figure S10) and an insertion was not called *in silico*, leaving its validation suggestive but inconclusive.

Discussion

By using LRS aligned to a recently updated human genome reference, we were able to reveal previously uncaptured structural and single-nucleotide variation across the β-globin locus in four African-ancestry populations with SCD. We were also able to leverage this approach to provide additional insights into the population genetics of this seminal locus and demonstrate its potential utility for exploring candidate disease-modifying variants. The complexity of the β-globin locus has been well established over decades of study; however, the utilization of newer sequencing technologies to reassess a locus of historical and clinical importance allowed us to characterize variation at a finer and more comprehensive scale than before.

One key finding from our study is that alignment to the existing hg38 genome reference for variant calling alone for these groups led to many likely false-positive variant calls. This is consistent with a growing acknowledgment of the limitations and "gaps" in the current reference, particularly for populations of African ancestry. By contrast, the T2T-CHM13.v2 genome reference was generated through the implementation of long-read technology similar to that used here, which facilitated a more complete sequence of the entire 70 kb β-globin locus—previous work demonstrated an up to 1.5-fold improved precision of variants identified in T2T-CHM13.v2 compared to hg38.[57] Accordingly, the T2T-CHM13.v2 alignment, which included an additional 1.1 kb of DNA, resolved many of the presumed false-positive calls from hg38, resulting in more robust SV calls. This additional DNA sequence also demonstrated additional SNVs, although most of these uncaptured SNVs were singleton events, most likely private to the individuals sequenced or resulting from PCR artifacts. Given recent expansions of SNV-aware short-read sequencing data from diverse populations,[4,58] the relative lack of uncaptured common SNVs in our African SCD cohort is perhaps unsurprising.

Our findings suggest that for populations of diverse, and particularly African, ancestries, aligning to the T2T-CHM13.v2 reference may be preferable for full variant discovery. Currently, however, contextual annotation of variants from T2T-CHM13.v2-aligned sequences necessitates translating from T2T-CHM13.v2 to hg38 coordinates, and for the T2T-CHM13.v2 sequence that is not represented in hg38, annotation is still in development (subjects and methods). For instance, in our dataset, there were ten structural and six SNVs with coordinates not represented in the hg38 reference; these were not included in our final analysis because of the uncertainty in location and difficulty in annotation. As human genetics moves toward a more complete reference, these limitations are likely to be quickly overcome, but in the short-term, individual groups will need to be cognizant of this limitation. In addition, most of our LRS data were generated using PacBio Sequel I, which has a high proportion of random sequence errors relative to more recent versions of the technology. We anticipated that many of these random errors would be identified and removed given the high sequence coverage of our PCR amplicons; however, this does not account for systematic errors or for failures of the PCR amplification itself and is subject to still-emerging computer calling algorithms. This is one reason for focusing our major observations on commonly observed events. Regardless, our results imply that LRS data generated using previous platforms could benefit from alignment to updated references.

The T2T-CHM13.v2 alignment is based predominantly on a single individual of South Asian ancestry; potentially, this inclusion of a non-European primary reference also aided in providing a broader reference for our locus. Relative to SV calls across the same region in African-ancestry individuals in AoU, we observed an increased number of calls in our SCD cohort of native Africans. Comparison with available AoU African-ancestry LRS callsets provided population context but was interpreted qualitatively primarily because the lack of overlap between the two callsets may reflect any of a combination of biological, technical, and analytical factors, including mismatched merging strategies, the inability to revisit read-level evidence from merged VCFs, unmodeled platform and coverage differences, and differences in sample ascertainment

between a general population cohort and a disease-enriched SCD cohort. We thus interpret this result with caution.

Therapeutics that target the β-globin locus are at the forefront of recent therapeutic advances for SCD; however, many of the ongoing and planned SCD clinical trials are predominantly undertaken in African Americans or Afro-Caribbeans, who represent a small subset of African ancestral genomes. Although our sample size is modest, our observation of multiple SVs in more than 50% of samples suggests that common SVs at this locus in individuals of African ancestry may be underrepresented in databases and argues for conducting similar LRS evaluations in larger native African populations. The importance of including ethnolinguistic diversity among our SCD samples from Africa, where more than 70% of individuals with SCD reside, was evident in the outsized uncaptured variant contributions from the Hausa from Nigeria. For example, uncaptured SNVs were only observed in a third of samples from the Igbo (also from Nigeria), but every SCD-affected individual identifying as Hausa from Nigeria contributed both uncaptured SNVs and SVs. Several uncaptured SVs were observed among individuals from Kenya, who were all from the Luo ethnolinguistic group (a distinct ethnolinguistic group from the Luhya represented in the 1000 Genomes Database). Several variants were almost exclusive to these Kenyan samples and would have been missed otherwise. Our study thus reinforces the efforts of the Human Pangenome Reference Consortium (HPRC)[57] and others to generate a reference that includes genomes from diverse populations,[57] even beyond 1000 Genomes cell lines, and suggests that such a reference, combined with LRS in diverse cohorts, could be particularly beneficial for understanding complex but functionally important loci such as the human leukocyte antigen (HLA) and KIR (killer-immunoglobulin region) loci. We also note that traditional RFLP sickle haplotypes do not reflect the breadth or depth of genomic variation evident in the β-globin cluster and are likely outdated for correlative clinical studies. Genetic studies of SCD will be better served by incorporating more comprehensive evaluations of *cis*-variation that can capture the complexity and granularity of genetic variation in these genomes.

We were able to identify a commonly occurring SV event that, although preliminary, is a suggestive candidate for native *cis*-acting regulation of HbF. The smallest region of overlap (∼4 kb) among the three individuals with above-baseline levels of HbF was found in the "proximal promoter" region of *HBB*, which has long been associated with increased HbF, including reports of hereditary persistence of HbF (HPFH; OMIM: 141749) among individuals with copy-number variants in this region. A recent study demonstrated a plausible mechanism of persistent HbF in this context that involves disruption of the *HBB* promoter with improved access of the LCR to the *HBG1* promoter, resulting in overexpression of *HBG* and elevated HbF levels.[59] The elevated HbF levels noted among individuals within our cohort with insertions in this region would be consistent with this mechanism. These insertions were observed among multiple individuals, suggesting that such events occur recurrently and relatively commonly at this site, making it an attractive locus for further study.

There are limitations and caveats to our study. First, our starting DNA was extracted from long-standing archived blood samples using standard protocols and was not the high-molecular-weight DNA typically recommended for LRS. The use of tiled amplicon PCR sequencing, while cost-effective, is a potential source of PCR artifacts and template biases that belies its utility for increasing read depth from aged DNA samples. This limitation was compounded by the high error rate of Sequel I, and together, these factors confound our attempts to orthogonally validate our candidate insertions.

Despite these limitations and the relatively small sample size employed, our study demonstrates the potential gains of increased inclusivity among research cohorts and highlights the potential for newer technologies to positively impact our understanding of well-established but genomically complex loci.

Data and code availability

The published article and supplemental information include the aggregate variant datasets generated or analyzed during this study. In-house scripts referenced in the manuscript and BED files for mapped CNVs/SVs are available through the β-globin GitHub repository (see web resources). Participant-level LRS reads and genotype data generated for this study have not been deposited in a public repository because the consent and ethics approvals for these human genomic data do not provide for unrestricted public release of potentially identifying individual-level data. Legitimate requests for de-identified population-genetic data will be accommodated by the corresponding author, subject to applicable institutional review board/ethics committee approvals and data-use agreements. AoU LRS data analyzed in this study are stored within a featured workspace on the National Institutes of Health's AoU Research Program Researcher Workbench. Access to the raw participant-level data and analysis code is restricted to authorized researchers and can be accessed via the AoU Researcher Workbench. Approved users wishing to re-analyze or replicate this work must satisfy the program's data access and ethics requirements by registering directly on the AoU Research Hub (https://www.researchallofus.org/). (see web resources).

Acknowledgments

This work utilized the computational resources of the National Institutes of Health (NIH) HPC Biowulf cluster (http://hpc.nih.gov/). We gratefully acknowledge AoU participants for their contributions, without whom this research would not have been possible. We also thank the NIH AoU Research Program for making available the participant data examined in this study. Portions of the work were supported by a seed grant from the

Caroline Wiess Law Fund for Research in Molecular Medicine at BCM to N.A.H. P.L. was supported by training grants 5-T32 GM 8307–25, 5-T32 GM 8307–27, and 5-T32 DK 60445–15 from the NIH to the Department of Molecular and Human Genetics at BCM. The efforts of N.A.H., Q.L., E.W.T., Y.H., and E.B. and portions of the reported work were supported by grant HG-200412 from the National Human Genome Research Institute (NHGRI) of the NIH. A.W. was supported by the National Heart Lung and Blood Institute (NHLBI; U24HL135600) of the NIH. M.M. and F.S. were supported by NHGRI grant 1U01HG011758-01. This work was supported in part by the Intramural Research Program of NHGRI, NIH. The work and views expressed in the manuscript do not necessarily reflect the views of BCM or the NIH.

Author contributions

N.A.H. conceived the study. M.M. and F.S. conducted the alignment and variant calling of structural variants and SNVs. A.R. and A.M.P. facilitated the T2T-CHM13.v2 reference mapping. B.T., R.C., T.A., G.A., S.O., A.W., and G.L. provided the samples and data for the individuals studied. N.J.H. and P.L. performed PCR-RFLP, sample QC, barcoding, and pre-sequence amplification. A.H., N.A., and E.W.T. conducted the ONT validation experiments. Y.H. analyzed the resulting ONT LRS data. Q.M. and S.J. performed the long-read PacBio sequencing. R.A.H. and K.C.W. assisted in the processing of PacBio data. Q.L. performed the AoU analyses. P.L. performed the primary data analyses and wrote the manuscript with E.B. and N.A.H. All co-authors read and approved the final submitted manuscript.

Declaration of interests

N.A.H. is a member of the advisory boards of *Cell* and *Cell Genomics*.

Web resources

All of Us Researcher Workbench, https://www.researchallofus.org/data-tools/workbench/
CHM13 GitHub, https://github.com/marbl/CHM13
DGV, http://dgv.tcag.ca/
GitHub, https://github.com/NHGRI/CCDGS-beta-globin
gnomAD, https://gnomad.broadinstitute.org/
Gnuplot, http://www.gnuplot.info/
GWAS Catalog, https://www.ebi.ac.uk/gwas/summary-statistics
OMIM, https://www.omim.org
Oxford Nanopore Technology, https://community.nanoporetech.com/docs/prepare/library_prep_protocols/premium-whole-genome-amplification-sqk-lsk110
LIMA, https://github.com/PacificBiosciences/barcoding#lima—demultiplex-pacbio-data
SAMtools, https://github.com/samtools/samtools

Supplemental information

Supplemental information can be found online at https://doi.org/10.1016/j.xhgg.2026.100669.

Received: April 6, 2026
Accepted: August 30, 2026

References

1. 1000 Genomes Project Consortium, Abecasis, G.R., Altshuler, D., Auton, A., Brooks, L.D., Durbin, R.M., Gibbs, R.A., Hurles, M.E., and McVean, G.A. (2010). A map of human genome variation from population-scale sequencing. Nature *467*, 1061–1073. https://doi.org/10.1038/nature09534.
2. 1000 Genomes Project Consortium, Auton, A., Brooks, L.D., Durbin, R.M., Garrison, E.P., Kang, H.M., Korbel, J.O., Marchini, J.L., McCarthy, S., McVean, G.A., and Abecasis, G.R. (2015). A global reference for human genetic variation. Nature *526*, 68–74. https://doi.org/10.1038/nature15393.
3. Wonkam, A., Munung, N.S., Dandara, C., Esoh, K.K., Hanchard, N.A., and Landoure, G. (2022). Five Priorities of African Genomics Research: The Next Frontier. Annu. Rev. Genomics Hum. Genet. *23*, 499–521. https://doi.org/10.1146/annurev-genom-111521-102452.
4. Choudhury, A., Aron, S., Botigué, L.R., Sengupta, D., Botha, G., Bensellak, T., Wells, G., Kumuthini, J., Shriner, D., Fakim, Y.J., et al. (2020). High-depth African genomes inform human migration and health. Nature *586*, 741–748. https://doi.org/10.1038/s41586-020-2859-7.
5. Gurdasani, D., Carstensen, T., Tekola-Ayele, F., Pagani, L., Tachmazidou, I., Hatzikotoulas, K., Karthikeyan, S., Iles, L., Pollard, M.O., Choudhury, A., et al. (2015). The African Genome Variation Project shapes medical genetics in Africa. Nature *517*, 327–332. https://doi.org/10.1038/nature13997.
6. Gurdasani, D., Carstensen, T., Fatumo, S., Chen, G., Franklin, C.S., Prado-Martinez, J., Bouman, H., Abascal, F., Haber, M., Tachmazidou, I., et al. (2019). Uganda Genome Resource Enables Insights into Population History and Genomic Discovery in Africa. Cell *179*, 984–1002.e36. https://doi.org/10.1016/j.cell.2019.10.004.
7. Kessler, M.D., Yerges-Armstrong, L., Taub, M.A., Shetty, A.C., Maloney, K., Jeng, L.J.B., Ruczinski, I., Levin, A.M., Williams, L.K., Beaty, T.H., et al. (2016). Challenges and disparities in the application of personalized genomic medicine to populations with African ancestry. Nat. Commun. *7*, 12521. https://doi.org/10.1038/ncomms12521.
8. Mills, M.C., and Rahal, C. (2020). The GWAS Diversity Monitor tracks diversity by disease in real time. Nat. Genet. *52*, 242–243. https://doi.org/10.1038/s41588-020-0580-y.
9. Sirugo, G., Williams, S.M., and Tishkoff, S.A. (2019). The Missing Diversity in Human Genetic Studies. Cell *177*, 1080. https://doi.org/10.1016/j.cell.2019.04.032.
10. Kachuri, L., Chatterjee, N., Hirbo, J., Schaid, D.J., Martin, I., Kullo, I.J., Kenny, E.E., Pasaniuc, B., Polygenic Risk Methods in Diverse Populations PRIMED Consortium Methods Working Group, Ge, T.G., and Witte, J.S. (2024). Principles and methods for transferring polygenic risk scores across global populations. Nat. Rev. Genet. *25*, 8–25. https://doi.org/10.1038/s41576-023-00637-2.
11. Sherman, R.M., Forman, J., Antonescu, V., Puiu, D., Daya, M., Rafaels, N., Boorgula, M.P., Chavan, S., Vergara, C., Ortega, V.E., et al. (2019). Assembly of a pan-genome from deep sequencing of 910 humans of African descent. Nat. Genet. *51*, 30–35. https://doi.org/10.1038/s41588-018-0273-y.
12. Adebamowo, C.A., Adeyemo, A., Ashaye, A., Akpa, O.M., Chikowore, T., Choudhury, A., Fakim, Y.J., Fatumo, S., Hanchard, N., Hauser, M., et al. (2022). Polygenic risk scores for CARDINAL study. Nat. Genet. *54*, 527–530. https://doi.org/10.1038/s41588-022-01074-3.

13. Kim, M.S., Naidoo, D., Hazra, U., Quiver, M.H., Chen, W.C., Simonti, C.N., Kachambwa, P., Harlemon, M., Agalliu, I., Baichoo, S., et al. (2022). Testing the generalizability of ancestry-specific polygenic risk scores to predict prostate cancer in sub-Saharan Africa. Genome Biol. *23*, 194. https://doi.org/10.1186/s13059-022-02766-z.
14. Adeyemo, A., Balaconis, M.K., Darnes, D.R., Fatumo, S., Granados Moreno, P., Hodonsky, C.J., Inouye, M., Kanai, M., Kato, K., Knoppers, B.M., et al. (2021). Responsible use of polygenic risk scores in the clinic: potential benefits, risks and gaps. Nat. Med. *27*, 1876–1884. https://doi.org/10.1038/s41591-021-01549-6.
15. Piel, F.B., Steinberg, M.H., and Rees, D.C. (2017). Sickle Cell Disease. N. Engl. J. Med. *377*, 305. https://doi.org/10.1056/NEJMc1706325.
16. Piel, F.B., Hay, S.I., Gupta, S., Weatherall, D.J., and Williams, T.N. (2013). Global burden of sickle cell anaemia in children under five, 2010-2050: modelling based on demographics, excess mortality, and interventions. PLoS Med. *10*, e1001484. https://doi.org/10.1371/journal.pmed.1001484.
17. Ogamba, C.F., Akinsete, A.M., Mbaso, H.S., and Adesina, O.A. (2020). Health insurance and the financial implications of sickle cell disease among parents of affected children attending a tertiary facility in Lagos, south-west Nigeria. Pan Afr Med J. *36*, 227. https://doi.org/10.11604/pamj.2020.36.227.24636.
18. Schroeder, W.A., Shelton, J.R., Shelton, J.B., Cormick, J., and Jones, R.T. (1963). The Amino Acid Sequence of the γ Chain of Human Fetal Hemoglobin. Biochemistry *2*, 992–1008. https://doi.org/10.1021/bi00905a016.
19. Stevens, M.C., Hayes, R.J., Vaidya, S., and Serjeant, G.R. (1981). Fetal hemoglobin and clinical severity of homozygous sickle cell disease in early childhood. J. Pediatr. *98*, 37–41. https://doi.org/10.1016/s0022-3476(81)80529-x.
20. Powars, D.R., Weiss, J.N., Chan, L.S., and Schroeder, W.A. (1984). Is There a Threshold Level of Fetal Hemoglobin That Ameliorates Morbidity in Sickle Cell Anemia? Blood *63*, 921–926. https://doi.org/10.1182/blood.V63.4.921.921.
21. Smith, E.C., and Orkin, S.H. (2016). Hemoglobin genetics: recent contributions of GWAS and gene editing. Hum. Mol. Genet. *25*, R99–R105. https://doi.org/10.1093/hmg/ddw170.
22. Uda, M., Galanello, R., Sanna, S., Lettre, G., Sankaran, V.G., Chen, W., Usala, G., Busonero, F., Maschio, A., Albai, G., et al. (2008). Genome-wide association study shows BCL11A associated with persistent fetal hemoglobin and amelioration of the phenotype of beta-thalassemia. Proc. Natl. Acad. Sci. USA *105*, 1620–1625. https://doi.org/10.1073/pnas.0711566105.
23. Cardoso, G.L., Diniz, I.G., Silva, A.N.L.M.d., Cunha, D.A., Silva Junior, J.S.d., Uchôa, C.T.C., Santos, S.E.B.d., Trindade, S.M.S., Cardoso, M.d.S.d.O., and Guerreiro, J.F. (2014). DNA polymorphisms at BCL11A, HBS1L-MYB and Xmn1-HBG2 site loci associated with fetal hemoglobin levels in sickle cell anemia patients from Northern Brazil. Blood Cells Mol. Dis. *53*, 176–179. https://doi.org/10.1016/j.bcmd.2014.07.006.
24. Lettre, G., Sankaran, V.G., Bezerra, M.A.C., Araújo, A.S., Uda, M., Sanna, S., Cao, A., Schlessinger, D., Costa, F.F., Hirschhorn, J.N., and Orkin, S.H. (2008). DNA polymorphisms at the BCL11A, HBS1L-MYB, and beta-globin loci associate with fetal hemoglobin levels and pain crises in sickle cell disease. Proc. Natl. Acad. Sci. USA *105*, 11869–11874. https://doi.org/10.1073/pnas.0804799105.
25. Wilber, A., Nienhuis, A.W., and Persons, D.A. (2011). Transcriptional regulation of fetal to adult hemoglobin switching: new therapeutic opportunities. Blood *117*, 3945–3953. https://doi.org/10.1182/blood-2010-11-316893.
26. Kim, J., Kang, J., Kim, Y.W., and Kim, A. (2021). The human β-globin enhancer LCR HS2 plays a role in forming a TAD by activating chromatin structure at neighboring CTCF sites. FASEB J. *35*, e21669. https://doi.org/10.1096/fj.202002337R.
27. Frangoul, H., Altshuler, D., Cappellini, M.D., Chen, Y.-S., Domm, J., Eustace, B.K., Foell, J., de la Fuente, J., Grupp, S., Handgretinger, R., et al. (2021). CRISPR-Cas9 Gene Editing for Sickle Cell Disease and β-Thalassemia. N. Engl. J. Med. *384*, 252–260. https://doi.org/10.1056/NEJMoa2031054.
28. Starlard-Davenport, A., Gu, Q., and Pace, B.S. (2022). Targeting Genetic Modifiers of HBG Gene Expression in Sickle Cell Disease: The miRNA Option. Mol. Diagn. Ther. *26*, 497–509. https://doi.org/10.1007/s40291-022-00589-z.
29. Ingram, V.M. (1957). Gene Mutations in Human Hæmoglobin: the Chemical Difference Between Normal and Sickle Cell Hæmoglobin. Nature *180*, 326–328. https://doi.org/10.1038/180326a0.
30. Pauling, L., Itano, H.A., Singer, S.J., and Wells, I.C. (1949). Sickle Cell Anemia, a Molecular Disease. Science *110*, 543–548. https://doi.org/10.1126/science.110.2865.543.
31. Holloway, K., Lawson, V.E., and Jeffreys, A.J. (2006). Allelic recombination and de novo deletions in sperm in the human beta-globin gene region. Hum. Mol. Genet. *15*, 1099–1111. https://doi.org/10.1093/hmg/ddl025.
32. Smith, R.A., Ho, P.J., Clegg, J.B., Kidd, J.R., and Thein, S.L. (1998). Recombination Breakpoints in the Human β-Globin Gene Cluster. Blood *92*, 4415–4421. https://doi.org/10.1182/blood.V92.11.4415.
33. Webster, M.T., Clegg, J.B., and Harding, R.M. (2003). Common 5' beta-globin RFLP haplotypes harbour a surprising level of ancestral sequence mosaicism. Hum. Genet. *113*, 123–139. https://doi.org/10.1007/s00439-003-0954-0.
34. Chakravarti, A., Buetow, K.H., Antonarakis, S.E., Waber, P.G., Boehm, C.D., and Kazazian, H.H. (1984). Nonuniform recombination within the human beta-globin gene cluster. Am. J. Hum. Genet. *36*, 1239–1258.
35. Hanchard, N.A., Rockett, K.A., Spencer, C., Coop, G., Pinder, M., Jallow, M., Kimber, M., McVean, G., Mott, R., and Kwiatkowski, D.P. (2006). Screening for recently selected alleles by analysis of human haplotype similarity. Am. J. Hum. Genet. *78*, 153–159. https://doi.org/10.1086/499252.
36. Ghansah, A., Rockett, K.A., Clark, T.G., Wilson, M.D., Koram, K.A., Oduro, A.R., Amenga-Etego, L., Anyorigiya, T., Hodgson, A., Milligan, P., et al. (2012). Haplotype analyses of haemoglobin C and haemoglobin S and the dynamics of the evolutionary response to malaria in Kassena-Nankana District of Ghana. PLoS One *7*, e34565. https://doi.org/10.1371/journal.pone.0034565.
37. Sedlazeck, F.J., Lee, H., Darby, C.A., and Schatz, M.C. (2018). Piercing the dark matter: bioinformatics of long-range sequencing and mapping. Nat. Rev. Genet. *19*, 329–346. https://doi.org/10.1038/s41576-018-0003-4.
38. Nurk, S., Koren, S., Rhie, A., Rautiainen, M., Bzikadze, A.V., Mikheenko, A., Vollger, M.R., Altemose, N., Uralsky, L., Gershman, A., et al. (2022). The complete sequence of a human genome. Science *376*, 44–53. https://doi.org/10.1126/science.abj6987.

39. Akingbola, T.S., Tayo, B.O., Salako, B., Layden, J.E., Hsu, L.L., Cooper, R.S., Gordeuk, V.R., and Saraf, S.L. (2014). Comparison of patients from Nigeria and the USA highlights modifiable risk factors for sickle cell anemia complications. Hemoglobin *38*, 236–243. https://doi.org/10.3109/03630269.2014.927363.
40. Tayo, B.O., Akingbola, T.S., Salako, B.L., McKenzie, C.A., Reid, M., Layden, J., Osunkwo, I., Plange-Rhule, J., Luke, A., Durazo-Arvizu, R., and Cooper, R.S. (2014). Vitamin D levels are low in adult patients with sickle cell disease in Jamaica and West Africa. BMC Hematol. *14*, 12. https://doi.org/10.1186/2052-1839-14-12.
41. Hanchard, N., Elzein, A., Trafford, C., Rockett, K., Pinder, M., Jallow, M., Harding, R., Kwiatkowski, D., and McKenzie, C. (2007). Classical sickle beta-globin haplotypes exhibit a high degree of long-range haplotype similarity in African and Afro-Caribbean populations. BMC Genet. *8*, 52. https://doi.org/10.1186/1471-2156-8-52.
42. Williams, L.M., Qi, Z., Batai, K., Hooker, S., Hall, N.J., Machado, R.F., Chen, A., Campbell-Lee, S., Guan, Y., Kittles, R., and Hanchard, N.A. (2018). A locus on chromosome 5 shows African ancestry-limited association with alloimmunization in sickle cell disease. Blood Adv. *2*, 3637–3647. https://doi.org/10.1182/bloodadvances.2018020594.
43. Mahmoud, M., Doddapaneni, H., Timp, W., and Sedlazeck, F.J. (2021). PRINCESS: comprehensive detection of haplotype resolved SNVs, SVs, and methylation. Genome Biol. *22*, 268. https://doi.org/10.1186/s13059-021-02486-w.
44. Li, H. (2018). Minimap2: pairwise alignment for nucleotide sequences. Bioinformatics *34*, 3094–3100. https://doi.org/10.1093/bioinformatics/bty191.
45. Smolka, M., Paulin, L.F., Grochowski, C.M., Horner, D.W., Mahmoud, M., Behera, S., Kalef-Ezra, E., Gandhi, M., Hong, K., Pehlivan, D., et al. (2023). Comprehensive Structural Variant Detection: From Mosaic to Population-Level. Preprint at bioRxiv. https://doi.org/10.1101/2022.04.04.487055.
46. Zheng, Z., Li, S., Su, J., Leung, A.W.S., Lam, T.W., and Luo, R. (2022). Symphonizing pileup and full-alignment for deep learning-based long-read variant calling. Nat. Comput. Sci. *2*, 797–803. https://doi.org/10.1038/s43588-022-00387-x.
47. Patterson, M., Marschall, T., Pisanti, N., van Iersel, L., Stougie, L., Klau, G.W., and Schönhuth, A. (2015). WhatsHap: Weighted Haplotype Assembly for Future-Generation Sequencing Reads. J. Comput. Biol. *22*, 498–509. https://doi.org/10.1089/cmb.2014.0157.
48. Danecek, P., Bonfield, J.K., Liddle, J., Marshall, J., Ohan, V., Pollard, M.O., Whitwham, A., Keane, T., McCarthy, S.A., Davies, R.M., and Li, H. (2021). Twelve years of SAMtools and BCFtools. GigaScience *10*, giab008. https://doi.org/10.1093/gigascience/giab008.
49. Rhie, A., Nurk, S., Cechova, M., Hoyt, S.J., Taylor, D.J., Altemose, N., Hook, P.W., Koren, S., Rautiainen, M., Alexandrov, I.A., et al. (2023). The complete sequence of a human Y chromosome. Nature *621*, 344–354. https://doi.org/10.1038/s41586-023-06457-y.
50. Zhao, H., Sun, Z., Wang, J., Huang, H., Kocher, J.P., and Wang, L. (2014). CrossMap: a versatile tool for coordinate conversion between genome assemblies. Bioinformatics *30*, 1006–1007. https://doi.org/10.1093/bioinformatics/btt730.
51. Wang, K., Li, M., and Hakonarson, H. (2010). ANNOVAR: functional annotation of genetic variants from high-throughput sequencing data. Nucleic Acids Res. *38*, e164. https://doi.org/10.1093/nar/gkq603.
52. Sollis, E., Mosaku, A., Abid, A., Buniello, A., Cerezo, M., Gil, L., Groza, T., Güneş, O., Hall, P., Hayhurst, J., et al. (2023). The NHGRI-EBI GWAS Catalog: knowledgebase and deposition resource. Nucleic Acids Res. *51*, D977–D985. https://doi.org/10.1093/nar/gkac1010.
53. MacDonald, J.R., Ziman, R., Yuen, R.K.C., Feuk, L., and Scherer, S.W. (2014). The Database of Genomic Variants: a curated collection of structural variation in the human genome. Nucleic Acids Res. *42*, D986–D992. https://doi.org/10.1093/nar/gkt958.
54. Cui, Y., Chen, X., Luo, H., Fan, Z., Luo, J., He, S., Yue, H., Zhang, P., and Chen, R. (2016). BioCircos.js: an interactive Circos JavaScript library for biological data visualization on web applications. Bioinformatics *32*, 1740–1742. https://doi.org/10.1093/bioinformatics/btw041.
55. Karczewski, K.J., Francioli, L.C., Tiao, G., Cummings, B.B., Alföldi, J., Wang, Q., Collins, R.L., Laricchia, K.M., Ganna, A., Birnbaum, D.P., et al. (2020). The mutational constraint spectrum quantified from variation in 141,456 humans. Nature *581*, 434–443. https://doi.org/10.1038/s41586-020-2308-7.
56. Akinsheye, I., Alsultan, A., Solovieff, N., Ngo, D., Baldwin, C.T., Sebastiani, P., Chui, D.H.K., and Steinberg, M.H. (2011). Fetal hemoglobin in sickle cell anemia. Blood *118*, 19–27. https://doi.org/10.1182/blood-2011-03-325258.
57. Wang, T., Antonacci-Fulton, L., Howe, K., Lawson, H.A., Lucas, J.K., Phillippy, A.M., Popejoy, A.B., Asri, M., Carson, C., Chaisson, M.J.P., et al. (2022). The Human Pangenome Project: a global resource to map genomic diversity. Nature *604*, 437–446. https://doi.org/10.1038/s41586-022-04601-8.
58. Byrska-Bishop, M., Evani, U.S., Zhao, X., Basile, A.O., Abel, H.J., Regier, A.A., Corvelo, A., Clarke, W.E., Musunuri, R., Nagulapalli, K., et al. (2022). High-coverage whole-genome sequencing of the expanded 1000 Genomes Project cohort including 602 trios. Cell *185*, 3426–3440.e19. https://doi.org/10.1016/j.cell.2022.08.004.
59. Topfer, S.K., Feng, R., Huang, P., Ly, L.C., Martyn, G.E., Blobel, G.A., Weiss, M.J., Quinlan, K.G.R., and Crossley, M. (2022). Disrupting the adult globin promoter alleviates promoter competition and reactivates fetal globin gene expression. Blood *139*, 2107–2118. https://doi.org/10.1182/blood.2021014205.

HGGA, Volume 7

Supplemental information

Mapping long-read sequences to a complete reference genome reveals uncaptured variants across the β-Globin cluster in Africans with sickle cell disease

Pamela Lurie, Medhat Mahmoud, Aparna Haldipur, Yixing Han, Qing Li, Edmond Wonkam Tingang, Natalie Asmus, Emilyn Banfield, Nancy J. Hall, Bamidele Tayo, Richard Cooper, Titilola Akingbola, George Ayodo, Stephen Obaro, Guillaume Lettre, Qing Meng, Shalini Jhangiani, R. Alan Harris, Kim C. Worley, Juyhun Kim, Arang Rhie, Adam M. Phillippy, Fritz Sedlazeck, Ambroise Wonkam, and Neil A. Hanchard

SUPPLEMENTAL MATERIALS AND METHODS

Supplemental Methods

Restriction Fragment Length Polymorphism Haplotyping

Briefly, five gene regions within the beta-globin cluster on chromosome 11 were amplified by PCR using GoTaq polymerase and buffer (Promega, USA), Deoxyribonucleotide triphosphate master mix;(Fermentas, USA), and forward and reverse primers (Eurofins MWG Operon) under the PCR cycling conditions given in **Table S2.** Amplified DNA (1ul) was then loaded onto a 2% agarose gel in 1X TAE buffer with 0.5 micrograms/mL ethidium bromide and run at 135V for 40 to 50 minutes before inspecting for the expected product band size. The appropriately amplified product was then digested according to the manufacturer's instructions by combining 10ul of DNA with a master mix containing the cut enzyme (Fermentas, USA) and Tango buffer (Fermentas, USA). The mix was left in the PCR thermocycler to incubate overnight at 37°C. Subsequently, the mix was immediately placed on ice until loaded into a 3% agarose gel with 1X TAE buffer with 0.5 micrograms/mL ethidium bromide and run at 160V for 40 – 50 minutes. Digestion products were scored as +/+ if both alleles were digested, as +/– if only one allele was digested (heterozygote), and as –/– if no digestion was noted. Samples were then classified as one of the common RFLP sickle haplotypes **(Table S2)**.

Supplemental Tables

Table S1 - Demographic distribution of SCD samples. ID - internal deidentified ID; Haplotypes - RFLP ascertained sickle haplotype; Origin - city or region of recruitment; *Louisiana, USA[41].

Country	Cases	Haplotypes	Ancestry Group	Origin	HbF
Nigeria	SC004	Benin	Yoruba	Ibadan	n/a
	SC006	Benin/Atypical	Yoruba	Ibadan	2.6
	SC026	Atypical	Yoruba	Ibadan	2.8
	347	Benin	Hausa		7.3
	467	Benin/Atypical	Hausa		11.2
	886	CAR/Benin	Hausa		26.4
	898	Benin	Hausa		1.3
	942	Benin	Hausa		5.6
	SC097	Benin	Igbo	Ibadan	6.8
	SC144	Benin	Igbo	Ibadan	5.2
	SC148	Benin	Igbo	Ibadan	1.6
Cameroon	476	Cameroon	Mbamois	Douala	0.8
	41	Benin	Bami	Douala	10.9
	15	Benin/Cameroon	bakoko/Dla	Douala	0.9
	21	Benin/Cameroon	Beti	Yaoundé	23.4
	38	Benin	Beti	MBALMAYO	13
	40	Benin/Cameroon	Beti	Yaoundé	12
	144	Benin	Beti	Yaoundé	n/a
Kenya	9215	CAR	Luo	Lwak	11.1
	9218	CAR	Luo	Lwak	2.3
	9220	CAR	Luo	Lwak	6.4
	9238	CAR	Luo	Lwak	3.1
	9239	CAR	Luo	Lwak	7.1
	9242	CAR	Luo	Lwak	2.6
	9250	Benin/Cameroon	Luo	Lwak	7.3
	9246	CAR	Luo	Lwak	6.1
	9258	CAR	Luo	Lwak	1.5
	9263	CAR/Cameroon	Luo	Lwak	6.6
	9287	CAR/Cameroon	Luo	Lwak	0.7
	9292	CAR	Luo	Lwak	6.2
	9299	CAR	Luo	Lwak	3.4
	9296	Senegal	Luo	Lwak	17.8
	9295	Senegal	Luo	Lwak	7.8
	2169	CAR	Luo	Lwak	12.5
USA	NR134	Senegal	English	Louisiana	n/a
	NR012	Senegal	English	Louisiana	n/a
	NR039	Senegal	English	Louisiana	n/a
	R014	CAR/Benin	English	Louisiana	n/a
	R116	CAR/Benin	English	Louisiana	n/a
	R26	Senegal	English	Louisiana	n/a

Table S2 - RFLP haplotype and thermal cycling parameters (upper) alongside expected cleavage patterns for RFLP haplotypes (lower).

GENE REGION	PRIMER SEQUENCE		PCR THERMAL CYCLING CONDITIONS	FRAGMENT SIZE (bp)
	FORWARD	REVERSE		
5'Gy	AACTGTTGCTTT ATAGGATTTT	TAGGAGCTTATTGA TAACCTCAGAC	1 cycle of 95 °C for 5min	657
			35 cycles of 94°C for 30sec, 53°C for 40sec, 72°C for 2min	
			1 cycle of 72°C for 7min<br>1 cycle of 4°C forever	
Gy/Ay1	TGCTGCTAATGC TTCATTACAA	AAGTGTGGAGTGTG CACATGA	1 cycle of 95 °C for 5min	780
			35 cycles of 94°C for 30sec, 53°C for 40sec, 72°C for 2min	
			1 cycle of 72°C for 7min<br>1 cycle of 4°C forever	
Gy/Ay2	TGCTGCTAATGC TTCATTACAA	TAAATGAGGAGCA TGCACACAC	1 cycle of 95 °C for 5min	780
			35 cycles of 94°C for 30sec, 53°C for 40sec, 72°C for 2min	
			1 cycle of 72°C for 7min<br>1 cycle of 4°C forever	
3'ψβ	TCTGCATTTGAC TCTGTTAGC	GGACCCTAACTGAT ATAACTA	1 cycle of 95 °C for 5min	614
			35 cycles of 94°C for 30sec, 55°C for 40sec, 72°C for 2min	
			1 cycle of 72°C for 7min<br>1 cycle of 4°C forever	
5'β	CTACGCTGACCT CATAAATG	CTAATCTGCAAGAG TGTCT	1 cycle of 95 °C for 5min	386
			30 cycles of 94°C for 30sec, 55°C for 40sec, 72°C for 40sec	
			1 cycle of 72°C for 7min<br>1 cycle of 4°C forever	

	XmnI<br>5'Gg	*HindIII*<br>Gg$_1$	*HindIIIA*<br>Gg$_2$	*HincII*<br>3b	*Hinfl*<br>5b
Senegal	+	+	-	+	+
Bantu/CAR	-	+	-	-	-
Cameroon	-	+	+	+	-
Benin	-	-	-	+	-
Arabic-Indian	+	+	-	+	-
Atypical	-	-	-	+	+

Table S3 - Long-read sequencing primers. Primers used to amplify beta-globin cluster for long-read sequencing.

	SEQUENCE	LEN	Tm	Dimer/ Secondary	GC%	E-VALUE	HG38 browser coordinates (Chr11)	Lift over from HG38 to T2T coordinates
AMP1	GTCAGGTGGTCAGCTTC (Forward)	17	56.4	No/No	58%	0.016	11:5280633-5280649	chr11:5339993-5340009
	TAAGTGCTCAGGGCTGTGTG (Reverse)	20	64.1	No/No	55%	0.008	11:5290195-5290214	chr11:5349548-5349567
AMP 2	GAGAGTCACCAGGTCAGT (Forward)	18	54.6	No/No	55%	0.083	11:5267198-5267215	chr11:5326554-5326571
	ATGAAGGCAGTGGCCCTT (Reverse)	18	64.7	No/Mod.	55%	0.083	11:5272969-5272986	chr11:5332331-5332348
AMP3	AATCTGCCATGGGAAGG (Forward)	17	61.1	No/No	53%	0.64	11:5257393-5257409	chr11:5316750-5316766
	TATGTCTTTCTCACCCCACCT (Reverse)	21	62.3	No/No	48%	0.042	11:5267310-5267330	chr11:5326666-5326686
AMP 4	ACGCTGATGCTGACTTGTGA (Forward)	20	64.8	No/No	50%	0.49	11:5252429-5252448	chr11:5311785-5311804
	CCCCTCAACATCACGTCCTT (Reverse)	20	66.2	No/No	55%	0.49	11:5257901-5257920	chr11:5317258-5317277
AMP10	ACCGGTCAAAGGCCATAGTG (Forward)	20	66	No/No	55%	0.016	11:5200307-5200326	chr11:5259660-5259679
	GTCTCATGCCAGAACCT (Reverse)	17	55.8	No/Weak	53%	0.64	11:5208290-5208306	chr11:5267638-5267654
AMP 5	CGCTCCAGGAAAAGTGACCT (Forward)	20	65	No/Weak	55%	0.008	11:5245566-5245585	chr11:5304931-5304950
	TTCTTCGTTGAGCCCCTTCC (Reverse)	20	67.5	No/No	55%	0.008	11:5252809-5252828	chr11:5312165-5312184
AMP 6	TAACATCTAGATTTCTGCACTGT (Forward)	20	57.2	No/Very Weak	35%	0.0004	11:5239005-5239027	chr11:5298378-5298400
	GACGAGGGGAGCTTTGCAAA (Reverse)	20	68.6	No/No	555	0.016	11:5247078-5247097	chr11:5306443-5306462
AMP 7	AGAGCTGCGGAGAAGAGGTA (Forward)	20	63.2	No/No	55%	0.008	11:5233089-5233108	chr11:5292462-5292481
	AGCACATAGATAGGCGGTG (Reverse)	19	60.3	No/Weak	53%	0.008	11:5240318-5240336	chr11:5299691-5299709
AMP 8	AGTTCCGGGAGACTAGCACT (Forward)	20	61.8	No/Very Weak	55%	0.008	11:5224887-5224906	chr11:5284255-5284274
	ACATGGGTATGGGAGAGGC (Reverse)	19	63.8	No/Very Weak	58%	0.021	11:5233652-5233670	chr11:5293025-5293043
AMP 9	TCGTGACTGAATTTTACCTT (Forward)	20	56.3	No/No	35%	0.008	11:5222580-5222599	chr11:5281948-5281967
	CCAGGCCATCACTAAAG (Reverse)	17	56.5	No/No	53%	0.33	11:5226663-5226679	chr11:5286031-5286047
AMP 11	CTACTGCCCAATCCTCCGTC (Forward)	20	65.8	No/No	60%	0.008	11:5595129-5595148	chr11:5654534-5654553
	ATAGTGAGGCGGCTCAGAGA (Reverse)	20	64	No/Mod.	55%	0.008	11:5603541-5603560	chr11:5662975-5662994

Table S4 - Barcode primers (Pacific Biosciences, California) for amplicon sequencing.

PRIMER ID	FORWARD SEQUENCE		PRIMER ID	REVERSE SEQUENCE
_0097_Forward	TACGATCGTAGCTGCT		_0097_Reverse	AGCAGCTACGATCGTA
_0098_Forward	TATACACACTCGCTCG		_0098_Reverse	CGAGCGAGTGTGTATA
_0099_Forward	AGCGCTGCGACACGCG		_0099_Reverse	CGCGTGTCGCAGCGCT
_0100_Forward	GTCGTAGCTGCTGTAT		_0100_Reverse	ATACAGCAGCTACGAC
_0101_Forward	CTGTACTAGAGCGTCT		_0101_Reverse	AGACGCTCTAGTACAG
_0102_Forward	TCGAGTGTATAGCTCA		_0102_Reverse	TGAGCTATACACTCGA
_0103_Forward	ACTGTGACAGTATGAT		_0103_Reverse	ATCATACTGTCACAGT
_0104_Forward	TGTCTGAGACGCATAC		_0104_Reverse	GTATGCGTCTCAGACA
_0105_Forward	CACTCACGTGTGATAT		_0105_Reverse	ATATCACACGTGAGTG
_0106_Forward	ATCGCATCGCAGAGAC		_0106_Reverse	GTCTCTGCGATGCGAT
_0107_Forward	TACTCATATATGCTAC		_0107_Reverse	GTAGCATATATGAGTA
_0108_Forward	GTCTACGCTCGTCGCG		_0108_Reverse	CGCGACGAGCGTAGAC
_0109_Forward	TGCGAGACTATCGCGA		_0109_Reverse	TCGCGATAGTCTCGCA
_0110_Forward	CAGATCTCTCTGATGT		_0110_Reverse	ACATCAGAGAGATCTG
_0111_Forward	GTAGAGTGATCGCGTC		_0111_Reverse	GACGCGATCACTCTAC
_0112_Forward	ACGACAGTCAGAGTAT		_0112_Reverse	ATACTCTGACTGTCGT
_0113_Forward	ATATATAGCTGATGCG		_0113_Reverse	CGCATCAGCTATATAT
_0114_Forward	TGCTATCTGAGATACT		_0114_Reverse	AGTATCTCAGATAGCA
_0115_Forward	CAGCAGATCATGTCGA		_0115_Reverse	TCGACATGATCTGCTG
_0116_Forward	TGCTGCGAGCGCTCTG		_0116_Reverse	CAGAGCGCTCGCAGCA
_0117_Forward	ACTATCGCAGCTCAGT		_0117_Reverse	ACTGAGCTGCGATAGT
_0118_Forward	CGTCTCTCGTCTGTGC		_0118_Reverse	GCACAGACGAGAGACG
_0119_Forward	GAGTCTCGATATACTA		_0119_Reverse	TAGTATATCGAGACTC
_0120_Forward	TGTCATGTGTACACAC		_0120_Reverse	GTGTGTACACATGACA

_0121_Forward	TCTGTCGATATACACT		_0121_Reverse	AGTGTATATCGACAGA
_0122_Forward	ACGTGCTCTATAGAGA		_0122_Reverse	TCTCTATAGAGCACGT
_0123_Forward	TATCAGCACGACATGC		_0123_Reverse	GCATGTCGTGCTGATA
_0124_Forward	GCTCTCACGATATCAG		_0124_Reverse	CTGATATCGTGAGAGC
_0125_Forward	TATATGCTCTGTGTGA		_0125_Reverse	TCACACAGAGCATATA
_0126_Forward	GATAGCTGCTAGCTGA		_0126_Reverse	TCAGCTAGCAGCTATC
_0127_Forward	TCTCATGTGTGAGCTA		_0127_Reverse	TAGCTCACACATGAGA
_0128_Forward	TCAGATGTGTCGCGAG		_0128_Reverse	CTCGCGACACATCTGA
_0129_Forward	CGTAGCTCAGACACTC		_0129_Reverse	GAGTGTCTGAGCTACG
_0130_Forward	TCAGAGACACTACGAG		_0130_Reverse	CTCGTAGTGTCTCTGA
_0131_Forward	ATCGAGCAGCAGTCGT		_0131_Reverse	ACGACTGCTGCTCGAT
_0132_Forward	CGTAGCTCGAGATGAG		_0132_Reverse	CTCATCTCGAGCTACG
_0133_Forward	GCTAGTCGATGACAGC		_0133_Reverse	GCTGTCATCGACTAGC
_0134_Forward	CATGATGCGAGACGCT		_0134_Reverse	AGCGTCTCGCATCATG
_0135_Forward	GTGTAGCGTAGACAGT		_0135_Reverse	ACTGTCTACGCTACAC
_0136_Forward	AGCACGTGTGTCGACA		_0136_Reverse	TGTCGACACACGTGCT

Table S5 - **Read lengths.** Raw read lengths and Canu trimmed contig read lengths per amplicon.

		Raw Reads		Canu contigs	
	expected length	avg read length	read length distribution	avg contig length	contig length distribution
amp1	9,581	6578	50 - 87967	7161.4	407 - 13917
amp2	5,788	6917	50 - 97123	6346.1	426 - 13995
amp3	9937	6942	50 - 72850	8076.7	439 - 18489
amp4	5,491	8301	50 - 76142	8207.1	412 - 22278
amp5	7,262	7017	50 - 101191	8088.8	416 - 21880
amp6	8,092	6972	50 - 86806	8410.7	414 - 24200
amp7	7,247	7876	50 - 76065	9018	444 - 29330
amp8	8,783	7687	50 - 116525	6899.3	471 - 8815
amp9	4,102	7463	50 - 72910	4100	424 - 10256

Table S7. Sample cohorts for all 2,800 participants with lrWGS data. Table adapted from All of Us user support hub online documentation Table 16, https://support.researchallofus.org/hc/en-us/articles/29475228181908-How-the-All-of-Us-Genomic-data-are-organized#01JJK689B1VNXW0KFJ3JC1X0N6. 42 CDRv8 participants were sequenced in two different sample batches.

**Cohort name**	**Sequencing facility**	**Sequencing platform**	**No. of samples**	**Samples of African ancestry**	**Minimum coverage**
HA_Seq_CDRv7	HA	PacBio Sequel IIe and Sequel II	1027	959	Mid-pass (12x)
HA_Rev_mid	HA	PacBio Revio	65		Mid-pass (12x)
BI_Seq_high	BI	PacBio Sequel IIe	84	16	High-pass (25x)
BI_Seq_mid	BI	PacBio Sequel IIe	198	46	Mid-pass (12x)
BI_Rev_mid	BI	PacBio Revio	803	191	Mid-pass (12x)
BCM_Seq_high	BCM	PacBio Sequel IIe	77	8	High-pass (25x)
BCM_Rev_high	BCM	PacBio Revio	111	10	High-pass (25x)
BCM_ONT_high	BCM	ONT R10.4 on PromethION	196	20	High-pass (25x)
JHU_ONT_high	JHU	ONT R10.4 on PromethION	128		High-pass (25x)
UW_Seq_high	UW	PacBio Sequel IIe	100		High-pass (25x)
UW_Rev_high	UW	PacBio Revio	53		High-pass (25x)
**Total samples**			**2842**	**1250**	

Table S9. Common structural variants (SV). CHR= chromosome; POS = reference start position in basepairs ((T2T.CHM13.v2); END =end position in basepairs; NUM = Number of individuals with called SV at the same location (SURVIVOR2). ANNO= nearest gene annotation in T2T.CHM13.v2; Conf SUPP = number of supporting genotype calls exactly matching SV call; AVE LEN= Average length; CI START | CI END = confidence intervals around potential start and end positions upstream (up-) and downstream (down-) relative to reference.

**CHR**	**POS**	**END**	**SV**	**ANNO**	**NUM**	**LEN (BP)**	**CI START (up-)**	**CI START (down-)**	**CI END** (up-)	**CI END** (down-)	**Conf SUPP**
chr11	5259660	5267655	DUP	*OR51V1*	6	7,995	-1	6	-2	8	6
chr11	5286643	5286643	INS	*HBB*	5	8,563	-1,805	4	-1,805	4	5
chr11	5293620	5293680	INV	*HBD*	9	75	0	571	-23	565	3
chr11	5295149	5295149	INS	Up- or in *HBD*	4	4,044	-219	0	-219	0	4
chr11	5297452	5297508	INV	Up- or in *HBD*	10	60	-2	260	-17	282	4
chr11	5299784	5299838	INV	Up- or in *HBD*	11	54	-76	0	-79	0	5
chr11	5317572	5317811	INV	Down- of *HBE1*	30	196	-470	960	-113	761	8
chr11	5324507	5324556	INV	Down- of *HBE1*	5	57	-592	0	-603	0	1
chr11	5330321	5330321	INS	*HBE1*	4	25,212	-1,889	0	-1,889	0	4
chr11	5341138	5341283	INV	*HBE1*	13	150	-305	0	-291	8	1
chr11	5340540	5348414	DEL	*HBE1*	17	-7,859	0	105	0	0	7
chr11	5343104	5343150	INV	*HBE1*	4	104	-883	0	-705	0	1
chr11	5345463	5345463	INS	*HBE1*	4	2,558	0	621	0	621	3
chr11	5346603	5346720	INV	*HBE1*	5	96	0	1,581	0	1,513	1

Supplemental Figures

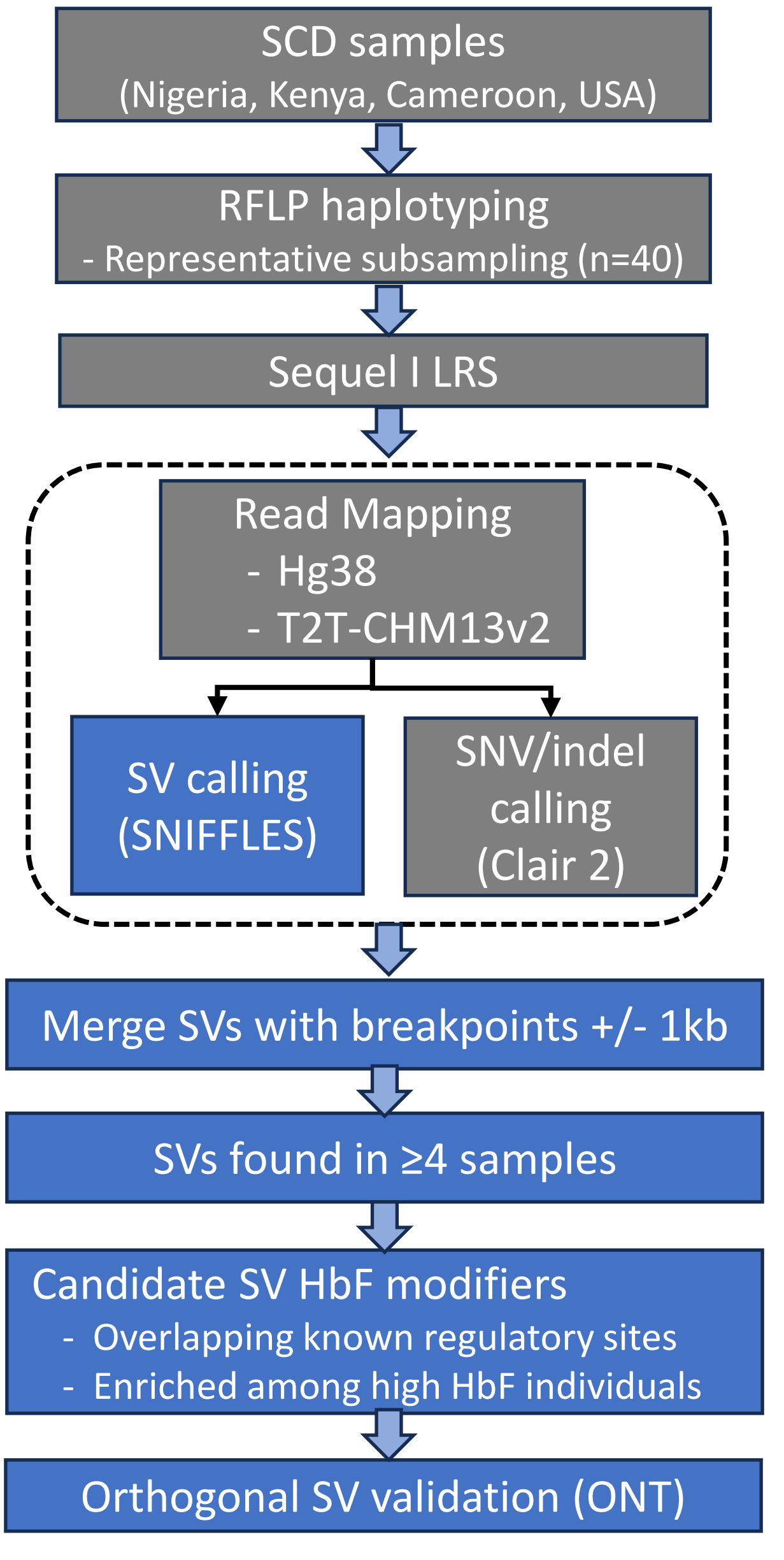

Figure S1. Study overview. DNA samples from SCD participants representative of classical SCD restriction fragment length (RFLP) haplotypes underwent long-read sequencing (LRS), prior to mapping reads to two human genome references and calling structural variants (SVs) and single nucleotide variants (SNVs) in PRINCESS (dotted line). SV calls were then prioritized for downstream evaluation.

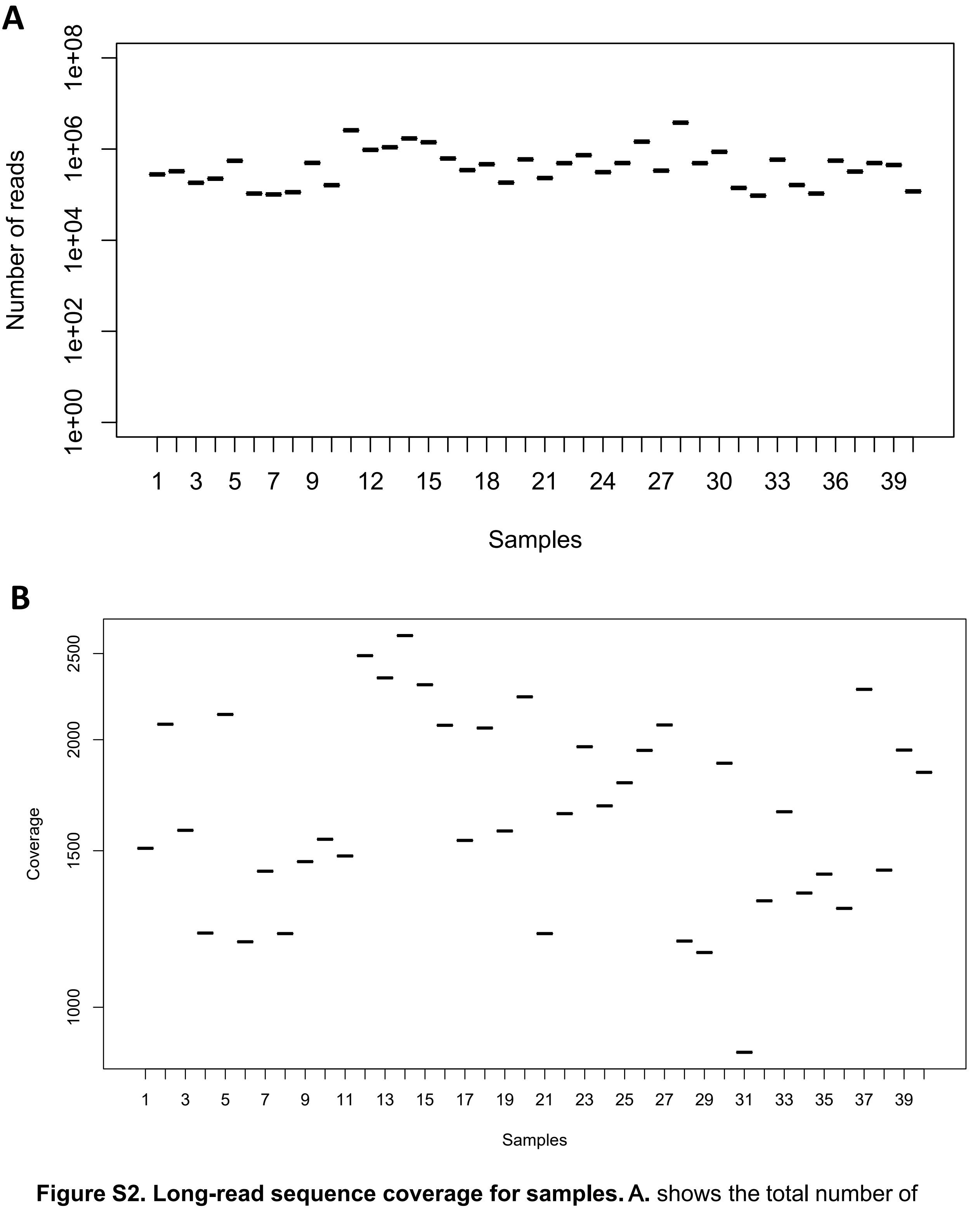

Figure S2. Long-read sequence coverage for samples. A. shows the total number of reads obtained for each sample. **B.** shows the median depth of coverage for each individual across the region.. Base pair coverage of the overall beta-globin locus in the T2T-CHM13.v2 reference genome ranged from +100bp - >90,000kb.

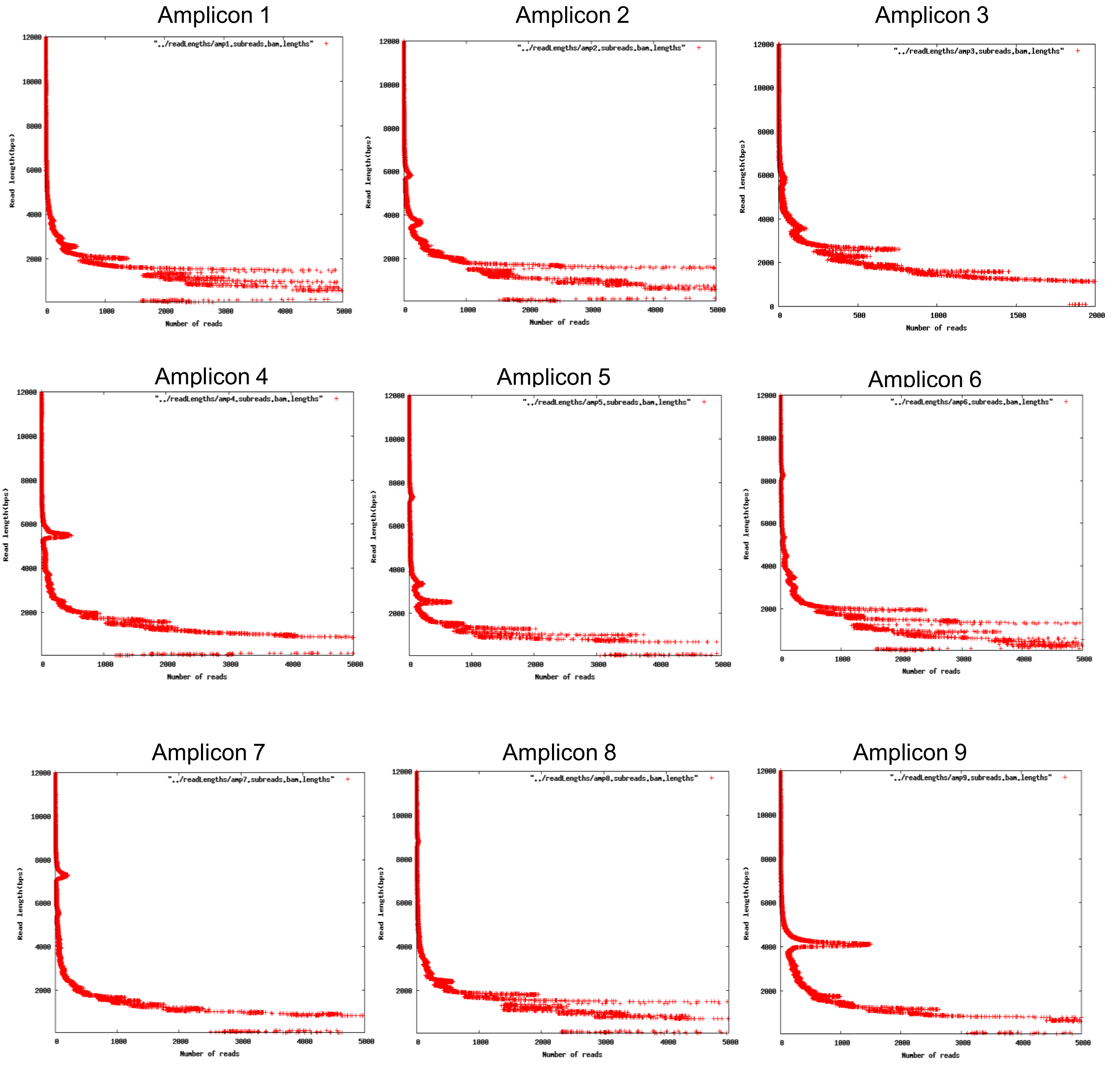

Figure S3. Distribution of raw reads for each gene-based amplicon sequenced.

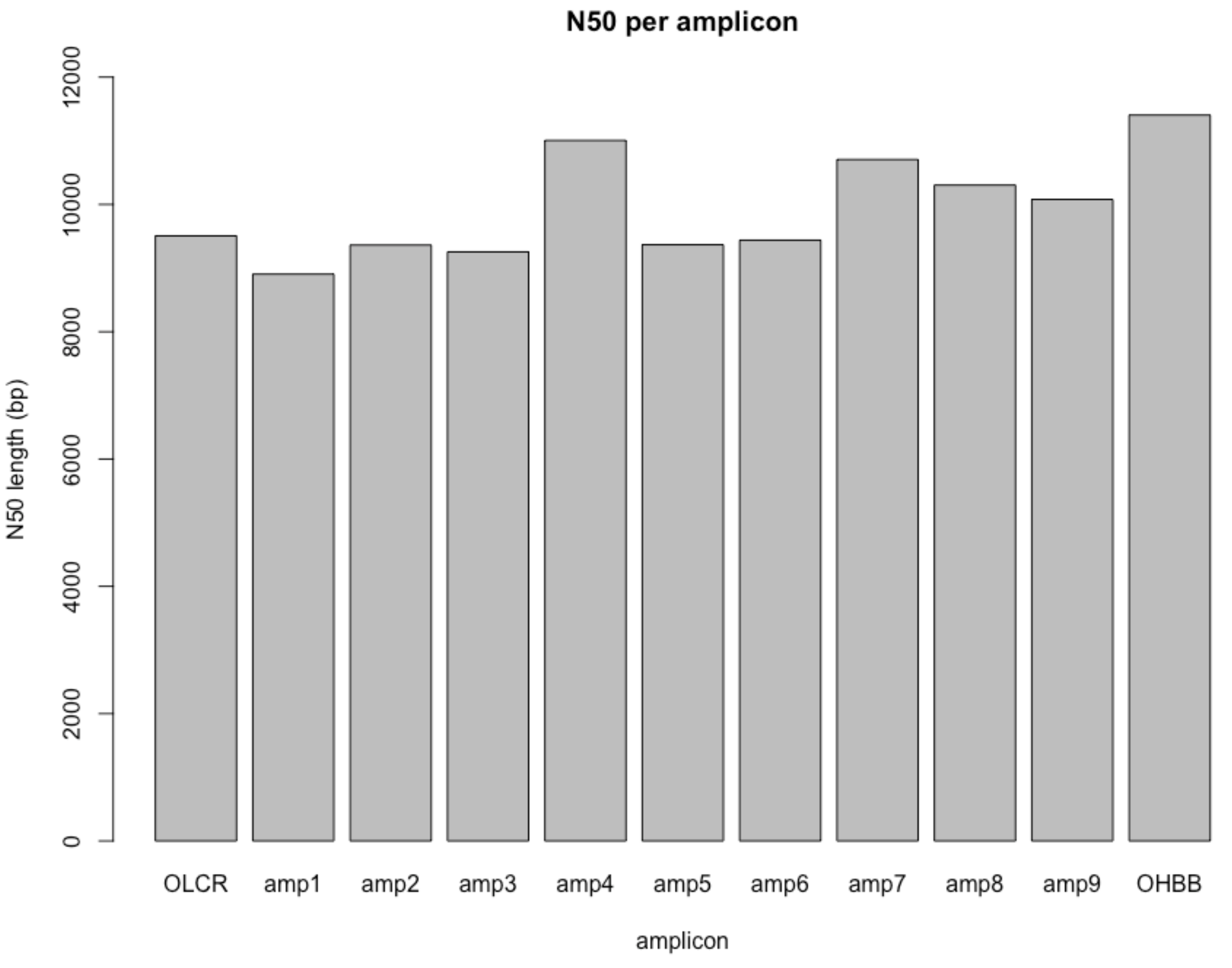

Figure S4. Read length N50 per amplicon from raw reads. Half of the amplicon is covered by reads larger than N50 length (expected read length exceeded).

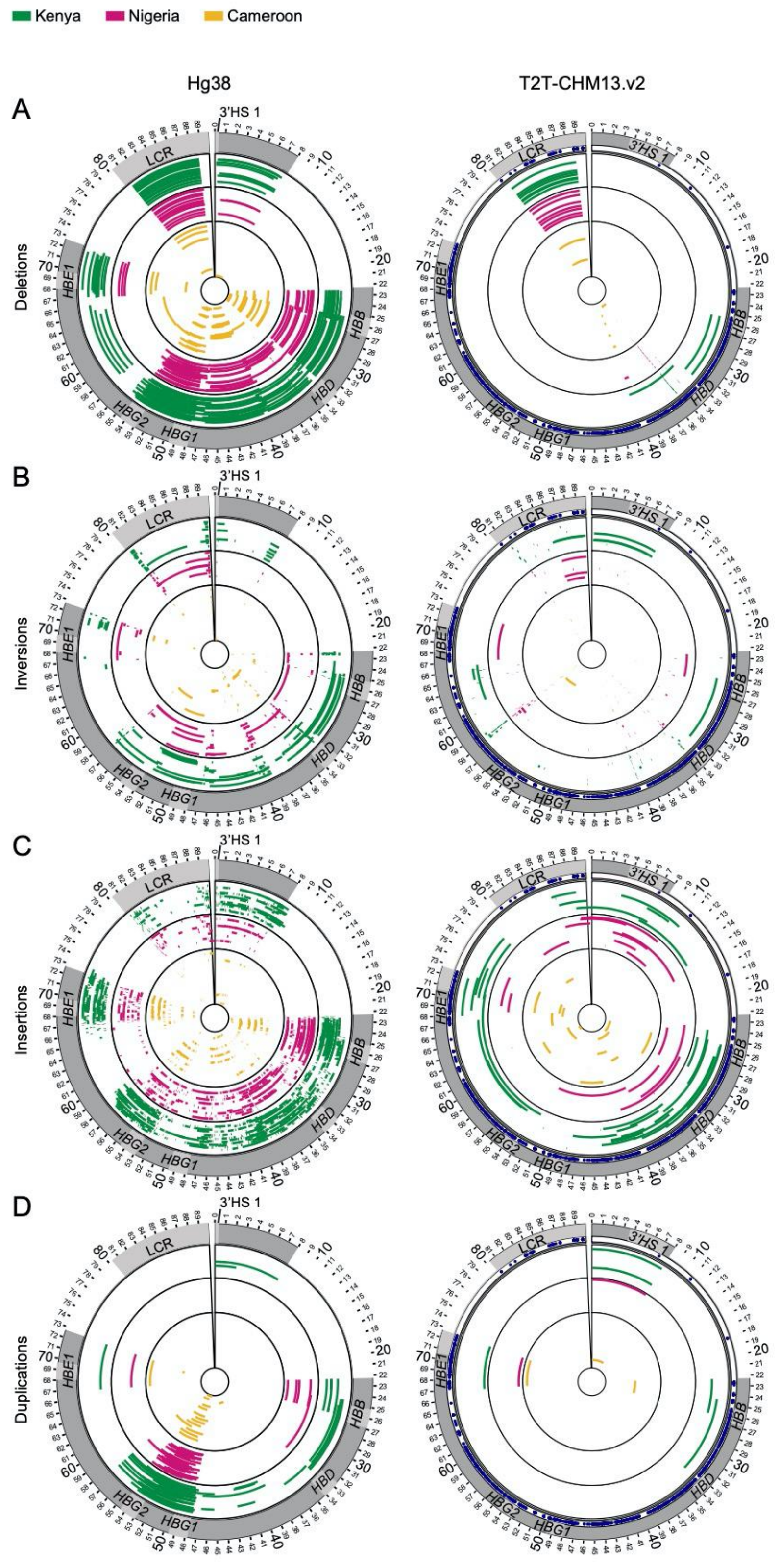

Figure S5. Genomic context of identified structural variants. Lighter gray shaded regions represent gene and Hypersensitive sites located in the beta-globin locus. Blue circles beneath the gene name and sequenced region track on T2T-CHM13.V2 circular plots represent areas of unalignment between the Hg38 and T2T-CHM13.v2 reference genomes. (**A**) Deletions. (**B**) Inversions. (**C**) Insertions. (**D**) Duplications. Geographical groups from Africa indicated by color: Kenya = green, Nigeria = pink, Cameroon = yellow. White spaces represent regions of the genome that was not sequenced. Note: 3' HS 1 location is further downstream *HBB* in T2T-CHM13.v2.

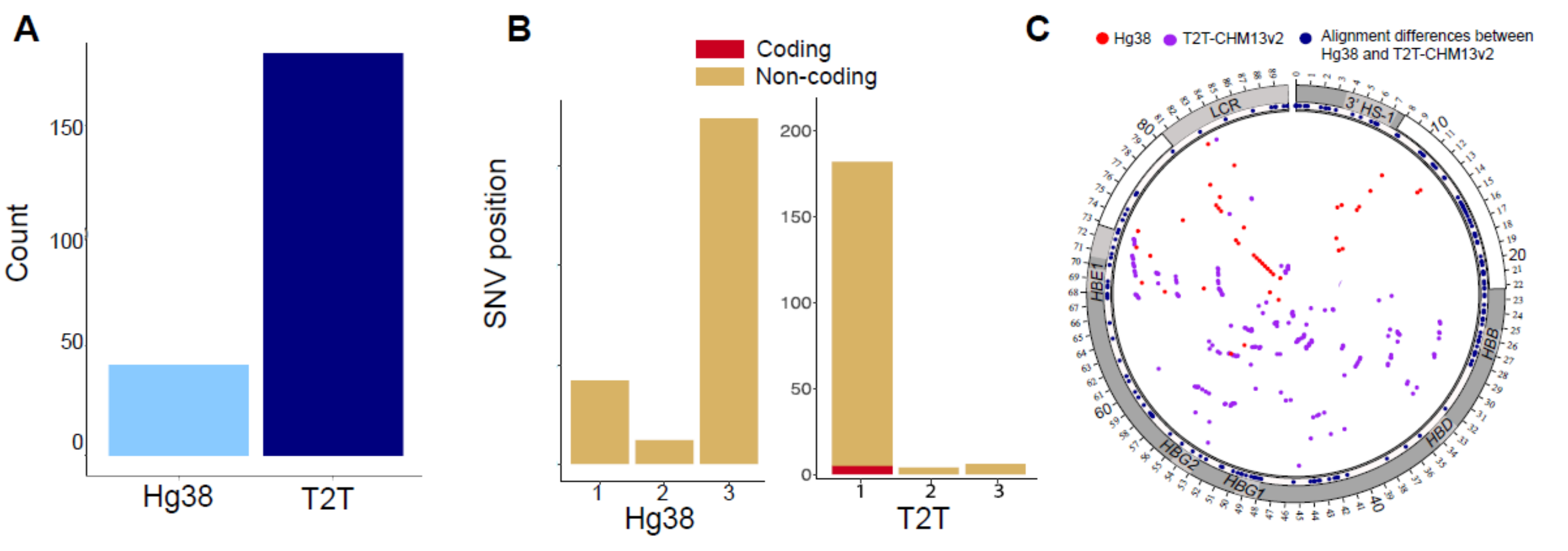

Figure S6. Single Nucleotide Variants (SNVs) in the beta-globin cluster of Africans with SCD. **A** - total number of novel SNVs called after aligning to either hg38 or T2T_CHM13.v2 (T2T) genome references. **B** – number of SNVs (X category) seen at each novel SNV position (Y axis); **C** – novel SNVs in genomic context across the beta-globin locus. Each concentric line from outside in represents an individual and each dot a different SNV in that individual. The outermost ring shows the relative genomic position in the region in kilobases (kbs). The next outermost ring illustrates non-coding (dark gray) or coding (lighter gray) targeted sequence; non-targeted regions are intervening in white. Navy blue dots in the next most outer ring represent DNA sequence found in T2T-CHM13.V2 but not in Hg38.

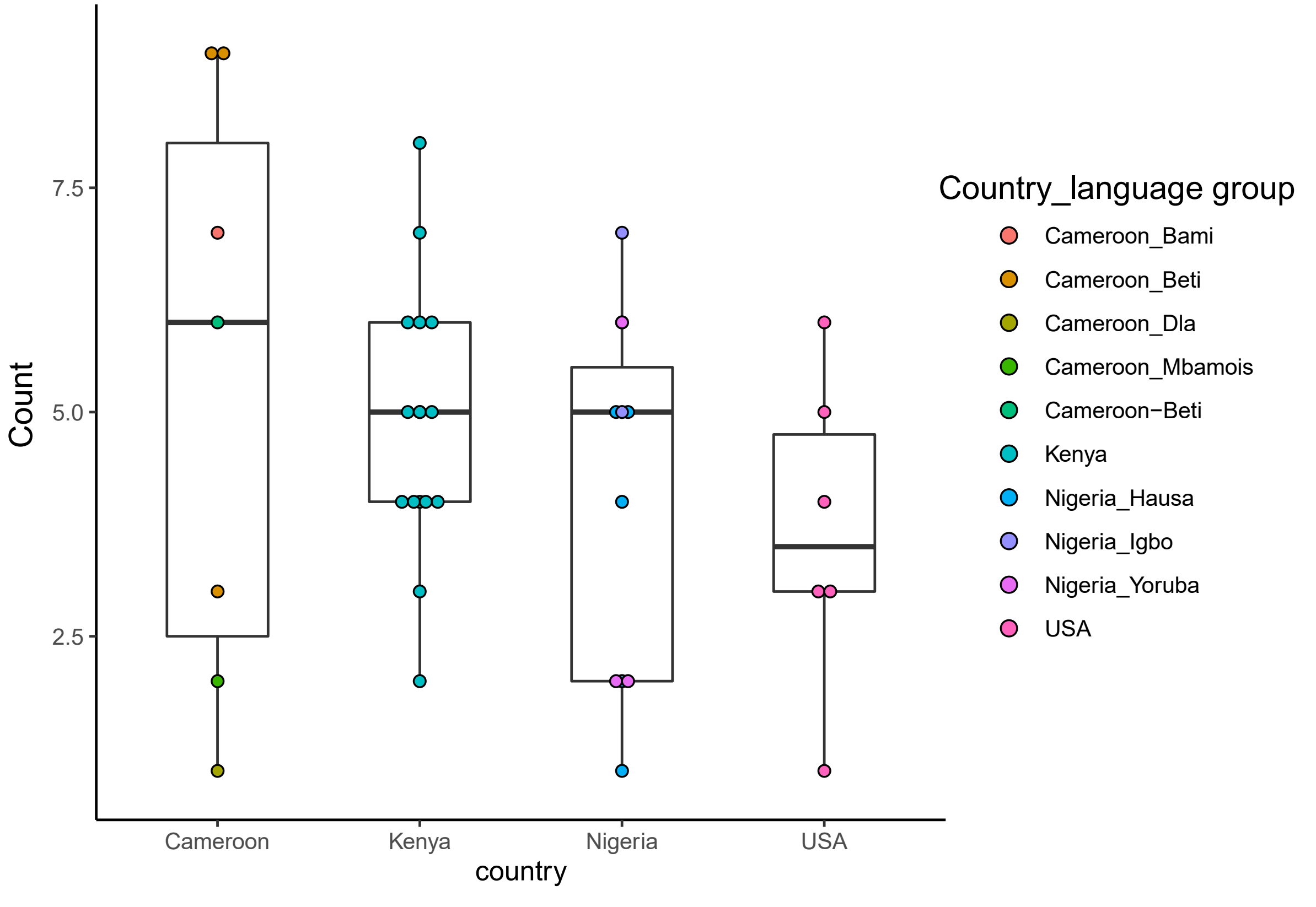

Figure S7. **Merged SVs per person grouped by geographical groups**. There is no significant difference between the median number of SVs per group

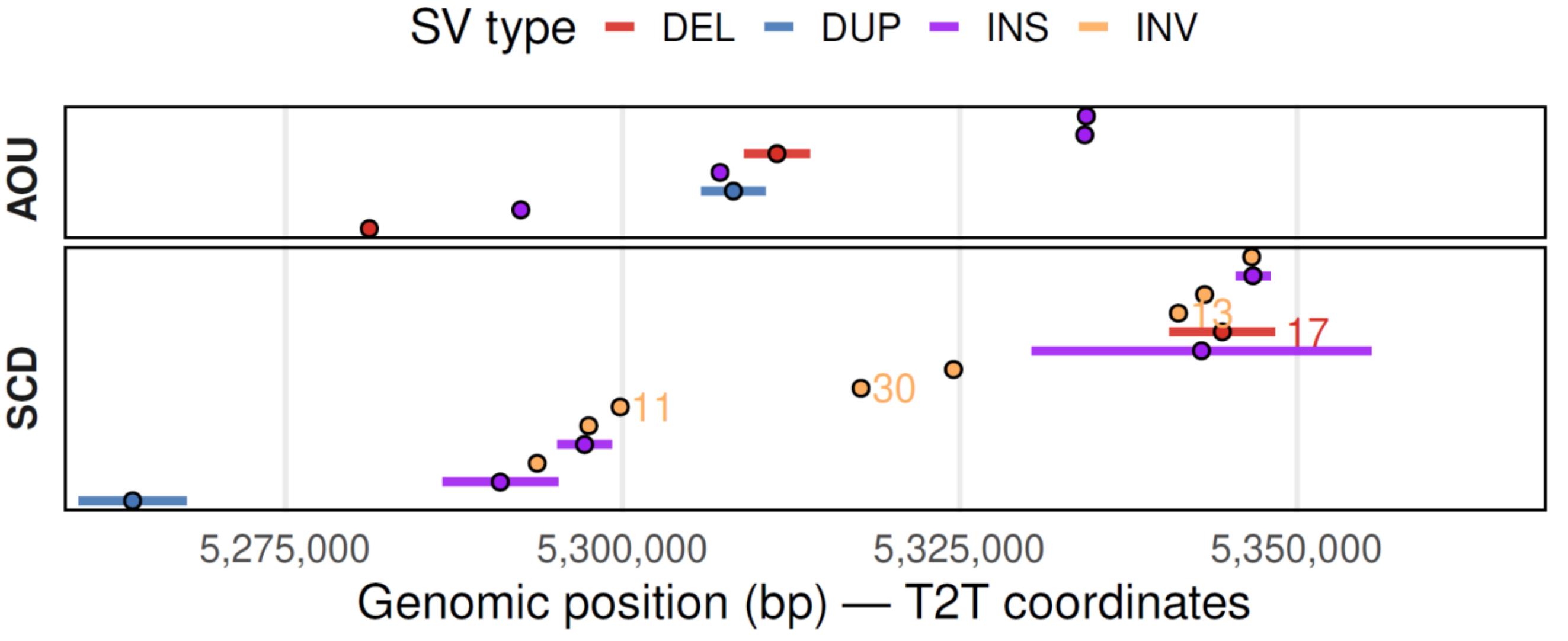

Figure S8. Structural variants (SVs) in African Sickle Cell Disease (SCD) participants and from long-read sequencing of All of Us (AoU) Africans. Numbers beside SVs are shown for SV shared by >10 samples. SV types – DEL=Deletion; DUP=Duplication; INS=Insertion; INV=inversion. Coordinates are referenced to T2T.CHM13v2.

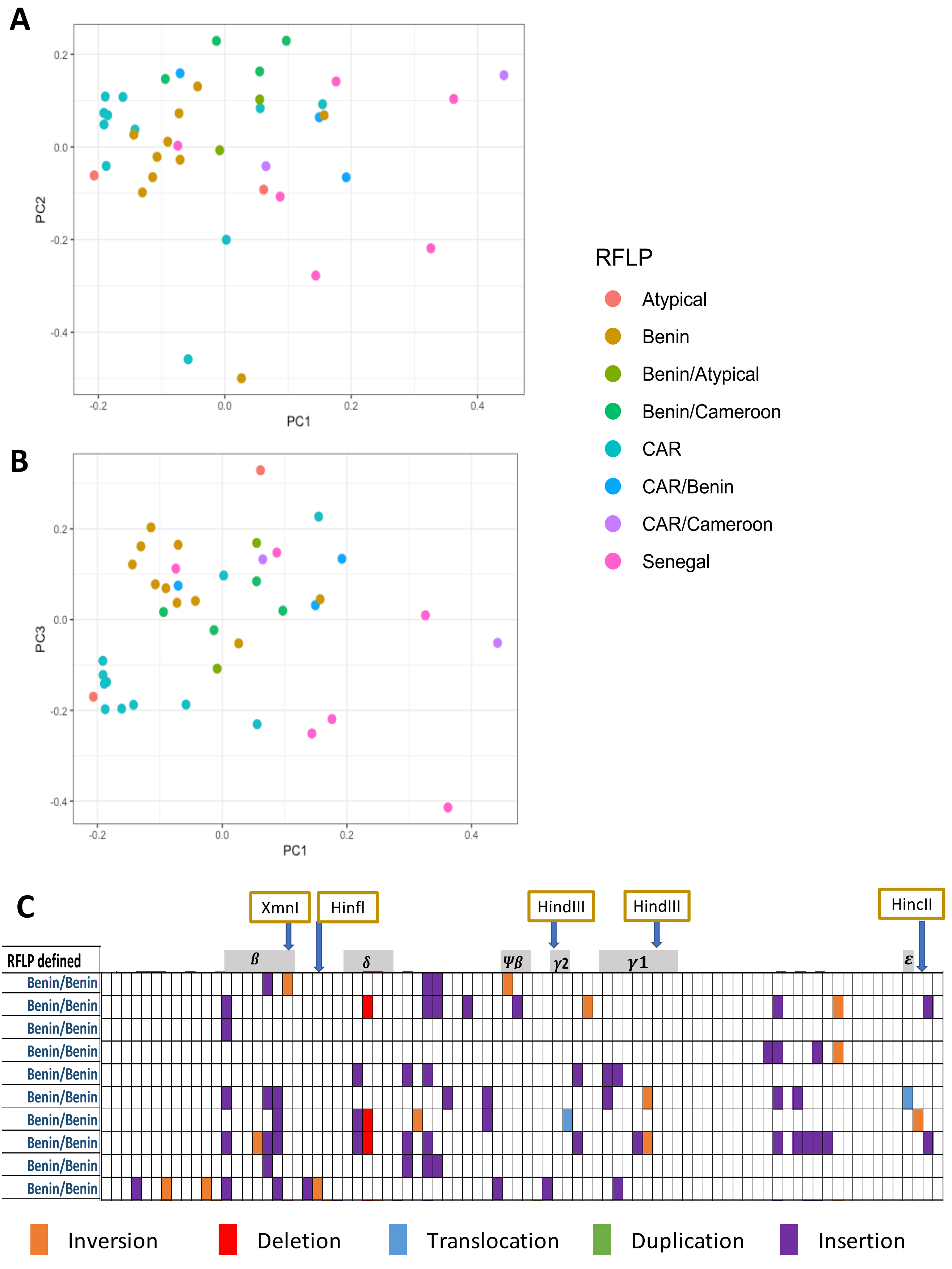

Figure S9. Beta-globin variation on RFLP defined haplotypes. A and **B** show principal components analysis of haplotype combinations by individual using LRS SVs. **C** shows SVs observed in individuals homozygous for the 'Benin' haplotype.

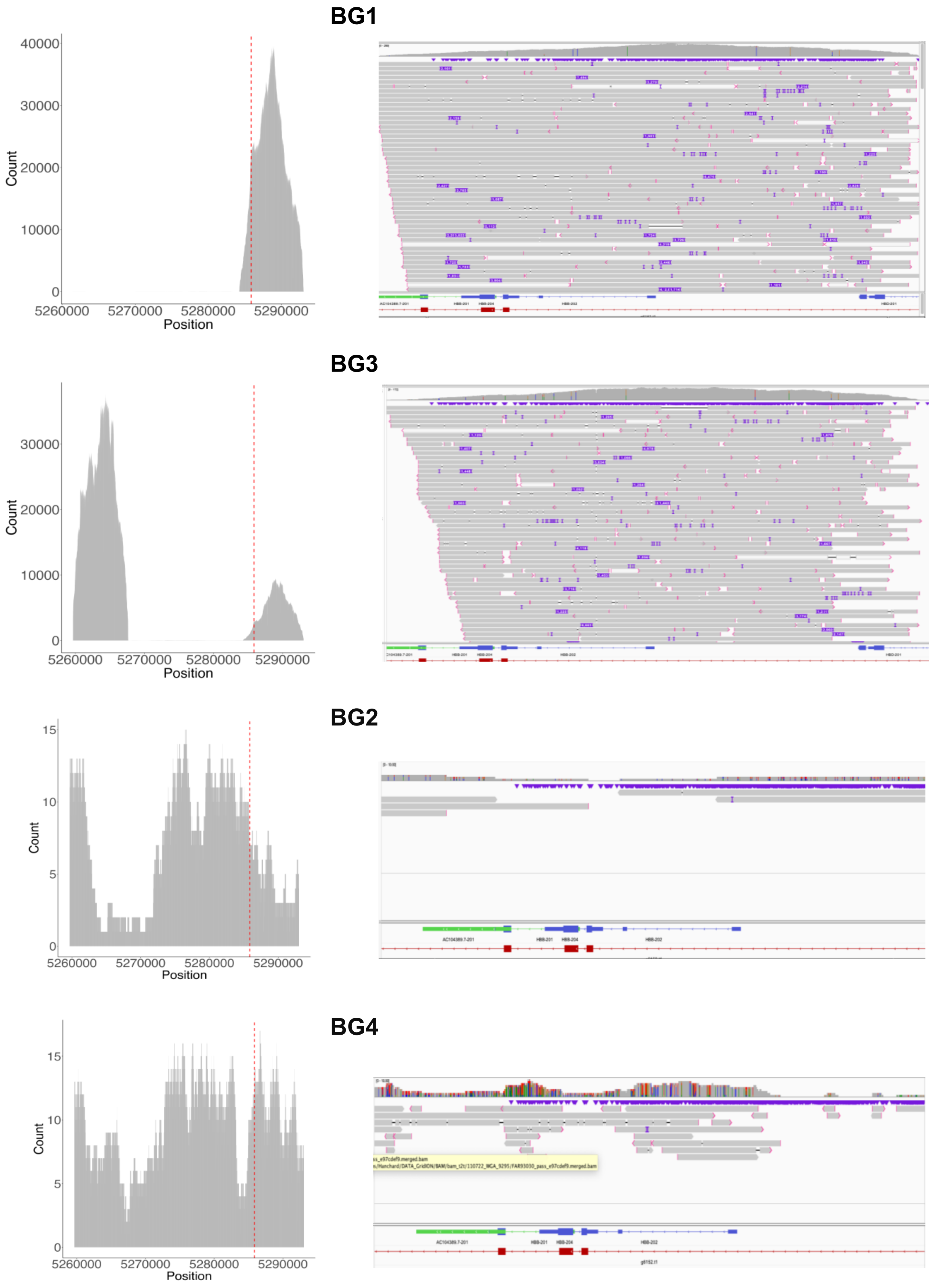

Figure S10. Oxford Nanopore Technology (ONT) validation sequencing (labelled BG1-BG4 in Figure 4). Left panels: Total sequence read coverage across a 30kb stretch on chromosome 11 including at *HBB* (dotted red line). The X-axis shows the genomic position using T2T-CHM13.v2 coordinates. The Y-axis shows the total count of ONT reads. Right panels: IGV visualization screenshots displaying sequencing reads with multiple insertions (purple bars) across *HBB* labelled in basepairs.
